# Pharmacogenetics in Response to Biological Agents in Inflammatory Bowel Disease: A Systematic Review

**DOI:** 10.3390/ijms26041760

**Published:** 2025-02-19

**Authors:** Octavio Ballesta-López, Mayte Gil-Candel, María Centelles-Oria, Juan Eduardo Megías-Vericat, Antonio Solana-Altabella, Hugo Ribes-Artero, Pilar Nos-Mateu, Javier García-Pellicer, José Luis Poveda-Andrés

**Affiliations:** 1 Pharmacy Department, Hospital Universitari i Politècnic La Fe, Av. Fernando Abril Martorell 106, 46026 Valencia, Spain; ballesta_oct@gva.es (O.B.-L.);; 2 Accredited Research Group on Pharmacy, Instituto de Investigación Sanitaria La Fe (IISLAFE), Av. Fernando Abril Martorell 106, 46026 Valencia, Spain; 3 Accredited Research Group on Hematology, Instituto de Investigación Sanitaria La Fe (IISLAFE), Av. Fernando Abril Martorell 106, 46026 Valencia, Spain; 4 Inflammatory Bowel Disease Unit, Gastroenterology Department, Hospital Universitari i Politècnic La Fe, Av. Fernando Abril Martorell 106, 46026 Valencia, Spain; 5 Management Department, Hospital Universitari i Politècnic La Fe, Av. Fernando Abril Martorell 106, 46026 Valencia, Spain

**Keywords:** inflammatory bowel disease, Crohn’s disease, ulcerative colitis, infliximab, adalimumab, vedolizumab, ustekinumab, polymorphism

## Abstract

Inflammatory bowel diseases (IBDs) are chronic inflammatory disorders influenced by microbial, environmental, genetic, and immune factors. The introduction of biological agents has transformed IBD therapy, improving symptoms, reducing complications, and enhancing patients’ quality of life. However, approximately 30% of patients exhibit primary non-response, and 50% experience a loss of response over time. Genetic and non-genetic factors contribute to variability in treatment outcomes. This systematic review aims to thoroughly analyze and assess existing studies exploring the relationships between genetic variations and individual responses to biologic drugs, in order to identify genetic markers that are predictive of treatment efficacy, risk of adverse effects, or drug toxicity, thereby informing clinical practice and guiding future research. PubMed and EMBASE papers were reviewed by three independent reviewers according to the Preferred Reporting Items for Systematic Reviews and Meta-Analyses [PRISMA] guidelines. Of the 883 records screened, 99 met the inclusion criteria. The findings of this review represent an initial step toward personalized medicine in IBD, with the potential to improve clinical outcomes in biological therapy.

## 1. Introduction

Inflammatory bowel diseases (IBDs) are chronic and inflammatory diseases that comprise two main forms: ulcerative colitis (UC) and Crohn’s disease (CD). Four key factors are known to play a predominant role in IBD development, such as luminal microbial antigens, environmental triggers, genetic susceptibility, and the immune response [1,2].

Initially, IBD therapy relied on aminosalicylates, antimetabolites (e.g., methotrexate, mercaptopurine, and azathioprine), and immunosuppressants (e.g., cyclosporine and corticosteroids). However, these treatments are associated with significant toxicity and therapeutic failure in a substantial proportion of patients [3,4,5].

A major advancement in the therapeutic landscape for IBD occurred with the introduction of monoclonal antibodies (mAbs) designed to inhibit the tumor necrosis factor-α (TNF-α) inflammatory pathway (e.g., infliximab (IFX), adalimumab (ADL), golimumab (GOL), and certolizumab pegol (CLZ)) in moderate and severe forms of IBD, in both induction and maintenance [6]. These agents have been shown to alleviate symptoms, reduce the risk of complications and surgery, and ultimately improve patients’ quality of life [7,8].

Nevertheless, IBD therapy remains challenging due to patients’ variability and therapeutic response. In fact, it has been noted that around 30% of patients with IBD show a primary non-response to anti-TNF therapies and, over time, around 50% of patients discontinue the treatment due to the loss of secondary response or even the appearance of adverse effects [9,10,11].

More recently, other mAbs with different mechanisms of action have been introduced. In particular, vedolizumab (VDZ) impedes the binding of α4β7-integrin expressed on memory T cells to the mucosal addressin cell adhesion molecule-1 (MadCAM-1). Ustekinumab (UST), for its part, inhibits the p40 subunit of IL-12 and IL-23; consequently, the subsequent inflammatory cascade is reduced. Additionally, the therapeutic landscape for UC has expanded, with a new class of drugs known as “small molecules” (e.g., tofacitinib, upadacitinib, and filgotinib), mainly targeting the Janus-Kinase (JAK) signaling pathway, as well as new and more innovative biologics or molecules that are being developed and approved year after year [12].

Although the exact reasons remain uncertain, it has been observed that a loss of response has been associated with a profusion of different mechanisms, including non-genetic (e.g., pharmacokinetic/pharmacodynamic processes, microbiological factors) or genetic factors that escape the “one-size-fits-all” paradigm [13,14].

Improvements in genetic characterization techniques and genome-wide association studies (GWASs) have made it possible to identify genetic variants that could influence the development of the disease, the response to treatment, and the development of adverse effects [14]. Although several studies have demonstrated an association between single-nucleotide polymorphisms (SNPs) and different pharmacological responses to treatments, some of the results appear to be controversial [15]. This systematic review aims to thoroughly analyze and assess existing studies that explore the relationship between genetic variations and individual responses to biologic drugs, in order to identify genetic markers that are predictive of treatment efficacy, risk of adverse effects, or drug toxicity, thereby informing clinical practice and guiding future research.

## 2. Materials and Methods

### 2.1. Literature Search Strategy

This systematic review was conducted by three independent reviewers, following the Preferred Reporting Items for Systematic Reviews and Meta-Analyses [PRISMA] guidelines [16]. The databases consulted included PubMed and EMBASE, with the search completed on 21 April 2023. Additionally, references from relevant studies and reviews were manually searched. The studies to be included in the review were selected independently by two authors [O.B.-L. and M.G.-C.]. Any discrepancies were resolved by a third reviewer [M.C.-O.]. Inter-reviewer agreement in study selection was excellent (kappa = 0.81).

Search terms were obtained from the Medical Subject Headings (MeSH) thesaurus developed by the US National Library of Medicine, as well as from additional relevant terms found in article titles and abstracts [tiab]. The final search strategy, constructed using Boolean connectors, was applied to the PubMed and EMBASE databases as follows: (IBD [tiab] OR inflammatory bowel disease [Mesh] OR Crohn disease [Mesh] OR CD OR [tiab] OR ulcerative colitis [Mesh] OR UC [tiab]) AND (pharmacogen* [tiab] OR polymorphism [tiab] OR SNP [tiab] OR single nucleotide polymorphism [tiab]) AND (adalimumab [Mesh] OR ustekinumab [Mesh] OR vedolizumab [Mesh] OR infliximab [Mesh] OR golimumab [Mesh] OR anti-TNF [tiab] OR tumor necrosis factor inhibitor [tiab]).

**Figure 1:**
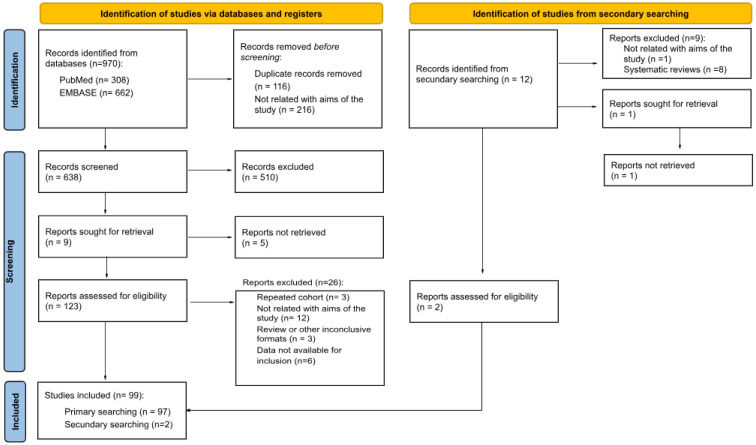
PRISMA flowchart showing the study selection process.

Duplicate articles were removed, and the remaining records were screened by title and abstract. The full texts were then reviewed to determine eligibility based on the inclusion and exclusion criteria (Figure 1).

### 2.2. Inclusion and Exclusion Criteria

The inclusion criteria encompassed observational studies with both retrospective and prospective designs, as well as relevant abstracts, with adult and pediatric patients of any gender and ethnicity, that analyzed the effects of genetic variants on the response to biological treatments in IBD.

Studies were excluded if they met any of the following criteria: (i) articles not specifically dedicated to the purpose of this study, such as reviews and systematic reviews, incomplete abstracts, and letters to the editor, (ii) articles written in a language other than English or Spanish, (iii) studies lacking information on the desired outcomes [genetic variant was not related to the treatment effect], (iv) studies not carried out on humans, and (v) studies providing duplicated information [abstracts that were subsequently published as a full paper].

### 2.3. Extraction of Relevant Data

For data extraction, the full text of the selected articles was obtained and studied by the three researchers, who independently extracted the relevant information using a standardized data extraction form, followed by a cross-check of the results. Disagreements between the reviewers were resolved through discussion among the three researchers.

The following data were extracted: gene and variant, study author, number of patients per diagnosis, age (range or mean), ethnicity (country), Hardy–Weinberg equilibrium (HWE), drug, clinical outcomes, statistical significance (HR/OR (95%CI) and/or *p*-value), and observations.

A follow-up literature search was conducted on 30 July 2024 to retrieve the most recent studies and provide updated results.

## 3. Results and Discussion

The search yielded 982 citations from databases and journals. Ultimately, 99 records fulfilled the inclusion criteria, and 883 studies were excluded (Figure 1). A total of 196 genes and 345 SNPs were identified in the included studies. The main data extracted from the selected studies are presented in Table 1, which is organized by genes and the variants studied. The table specifies the study author(s), number of patients per diagnosis, age (mean or median), ethnicity (country), whether HWE was met, the drug studied, and clinically significant outcomes, along with the corresponding statistical parameters. Refer to Supplementary Table S1 for results showing no statistically significant differences.

Key findings suggest that the genetic polymorphisms studied may significantly influence clinical outcomes. Clinical outcomes were categorized into the following groups: response (including improvement, beneficial effects, increase in responders, and clinical remission), non-response (including non-response, lack of response, and refractory cases), persistence of response (including time to loss of response, time to failure, long-term response, protection, and loss of response), higher/lower trough level (TL), and development of anti-drug antibodies (ADA) or adverse events.

### 3.1. Response and Non-Response to Anti-TNF Therapy

Certain genetic variants demonstrate favorable responses to anti-TNF agents. For instance, the variant rs10210302 (T allele) in the autophagy-related 16-like 1 (*ATG16L1*) gene was associated with an improved treatment response (OR: 9.44 (2.49–35.83), *p* < 0.001) [17]. Similar associations were observed in the *IL1B* gene with the rs4848306 (A allele) variant (OR: 1.85 (1.05–3.27), *p* = 0.03) [18]. Likewise, in the *IL17A* gene, the rs2275913 variant AA genotype was correlated with a positive response in CD (HR: 0.320 (0.111–0.920), *p* = 0.034) [19]. Additionally, variants in the *TNFRSF1A* gene, specifically rs4149570 (OR: 2.07 (1.03–4.15), *p* = 0.04 for IBD; and OR: 1.92 (1.02–3.60), *p* = 0.04 for CD) [18,20], and variants in the *TNFRSF1B* gene, including rs3397 (CC genotype) (OR: 3.19 (0.95–16.78), *p* = 0.05) and rs1061622 (G allele) polymorphisms (OR: 4.2 (1.2–18.2), *p* = 0.014) [21,22], indicated a better therapeutic response. These findings underscore the relevance of TNF receptor polymorphisms as significant markers for potential clinical benefit.

Variants linked to reduced or absent response to anti-TNF drugs suggest significant contraindications. In the *ATG16L1* gene, the rs2241880 (T allele) variant, along with the *IL17A* gene variant rs2275913 (A allele), showed associations with non-response (HR: 2.8 (1.0–8.7), *p* = 0.048; [23] and OR: 0.42 (0.18–1.00), *p* = 0.05 [18], respectively). Similarly, in the *C1orf106* gene’s rs61740234 variant, the CC and TT genotypes were correlated with non-responsiveness (OR: 4.49 (1.31–15.32), *p* = 0.010) [24]. Additionally, in the *CD14* gene, the rs2569190 variant showed that the presence of the A allele was linked to a reduced likelihood of response (OR: 0.54 (0.3–0.98), *p* = 0.04) [18]; however, another study highlighted the G allele as being associated with non-responsiveness to ADL (*p* = 0.026) [25]. The *FAS* gene polymorphism rs7896789 (C allele and CC genotype) has also been associated with a lack of response in CD patients (OR: 15.22, *p* = 0.003 and OR: 3.63 (1.261–10.425), *p* = 0.03) [26,27]. Similarly, the Fas ligand (*FASLG*) gene variant rs763110 was associated with poorer responses, with significant associations found between the CT or CC genotypes and non-response to IFX (OR: 0.11 (0.08–0.56), *p* = 0.002 and OR: 4.30 (1.45–12.80), *p* = 0.009) [28,29], suggesting that individuals with these genotypes may have an elevated risk of treatment failure. Similarly, for the *IL1RN* gene, the polymorphism rs4251961—specifically, the C allele—was also associated with non-response (OR: 0.42 (0.18–0.98), *p* = 0.04 for UC; and OR: 0.81 (0.66–1.00), *p* = 0.049 for IBD [18,20]. In the IL1B gene variant rs1143634, the C allele has been associated with non-response in CD patients (*p* = 0.027) [30]. In contrast, another study identified the T allele as being associated with an increased likelihood of non-response (OR: 2.59 (1.093–6.113), *p* = 0.03) [27]. Further support comes from NFκB pathway polymorphisms, with variants in the *TLR2* gene (rs11938228), the *TLR4* gene (rs1554973), and the *TLR9* gene (rs352139) all correlating with reduced response, suggesting an inflammatory signaling pathway link to treatment resistance [18,20,25].

### 3.2. Persistence of Response

Markers of sustained anti-TNF response are especially valuable in identifying candidates that are likely to maintain long-term efficacy. The *ATG16L1* gene variant rs12994997 (G allele) (OR: 0.68, *p* = 0.05) [31], the *IL6* gene variant rs10499563 (C allele) (HR 0.2 (0.05–1), *p* = 0.05), and the *LY96* gene variant rs11465996 (C allele) (HR 5.5 (1.1–28.1), *p* = 0.042) [32] have all shown positive correlations with treatment persistence. Furthermore, the *NOD2* gene’s rs5743289 variant (OR: 1.79, *p* = 0.033) [33] and the *TNFRSF1B* gene’s rs1061624 variant (A allele) (HR: 0.041 (0.18–0.92), *p* = 0.03) [34] were associated with sustained response, which may help in therapeutic decision-making for patients requiring long-term treatment.

In contrast, the *ATG16L1* gene variant rs2241880 (AA genotype) has been linked with a loss of response to IFX (OR: 0.551 (0.319–0.951) *p* = 0.030 for IBD; OR: 0.320 (0.125–0.822) *p* = 0.012 for UC) [35].

### 3.3. Trough Levels 

Polymorphisms affecting IFX TLs contribute to variability in drug bioavailability. Lower TLs were observed in patients with the *ATG16L1* gene variants rs7587051 and rs143063741 (GG genotypes) (OR: 1.65 (0.87–6.22), *p* = 0.015 and OR: 2.93 (1.31–5.40), *p* < 0.001, respectively) [36]. Similarly, the *C1orf106* gene variant rs442905 (GA genotype) was linked to reduced TLs (2.59 µg/mL [95% CI, 1.26–5.59 µg/mL] vs. 3.68 µg/mL [95% CI, 1.79–6.09 µg/mL, *p* = 0.046) [36]. Likewise, the C allele (rs763110) in the *FASLG* gene was associated with lower drug TLs (*p* = 0.032) [32], which also impacts the effectiveness. The *NOD2* gene’s combined variants rs2066844, rs2066845, and rs2066847 were also associated with reduced TLs (*p* = 0.016; IFX *p* = 0.038; ADL *p* = 0.033) [37], reflecting how genetic interactions can influence drug exposure.

In contrast, the *C1orf106* gene variant rs59457695 (CC genotype) was associated with higher IFX TLs (3.67 µg/mL [95% CI, 1.75–5.95 µg/mL] vs. 1.97 µg/mL [95% CI, 1.03–5.15 µg/mL, *p* = 0.022) [36]. In the same way, the *IL6* gene’s rs10499563 variant (CC genotype) demonstrated a significant correlation with higher TLs (OR: 21.43 (2.34–196.43), *p* = 0.007) [34], indicating potentially beneficial pharmacokinetic responses.

### 3.4. ADA Development and Immunogenicity

Anti-TNF immunogenicity can be potentiated by specific genetic profiles, leading to ADA development and reduced therapeutic efficacy. Notably, the rs396991 variant of the *FCGR3A* gene (*p* = 0.004 at week 14, *p* = 0.001 at week 22, and *p* = 0.01 at week 52 [38]; OR: 2.94 (1.24–6.96), *p* = 0.01 [39]; *p* = 0.004 [40]) has emerged as a consistent indicator of heightened ADA risk. In the same way, the rs3397 variant (CC genotype) in the *TNFRSF1B* gene was associated with ADA formation (OR: 0.23 (0.06–0.94), *p* = 0.041) [41]. The HLA-DQA1*05 (rs2097432) variant has been identified as a predictor of immunogenicity and secondary loss of response to TNF-α compared with non-carriers of this variant [39,42,43,44,45]. Sazonovs et al. (2020), in a brief abstract, identified a statistically significant association between this genetic variant and the development of ADA in a cohort of 1240 patients, with an HR of 1.90 (*p* = 5.88 × 10^−13^) [43]. In the same way, Zhu et al. (2023) found that HLA-DQA1*05 carriers had a markedly higher incidence of ADA development [39]. In other studies, such as those of Cheli et al. (2023) and Hu et al. (2021), no significant differences between carriers and non-carriers were observed [41,46], particularly in settings where proactive therapeutic drug monitoring (TDM) was routinely performed to maintain target serum drug concentrations.

### 3.5. Adverse Events

Pharmacogenetic markers predicting adverse events can improve safety in anti-TNF treatment. For instance, the *FASLG* gene’s rs763110 T allele is correlated with increased adverse events during IFX treatment (OR: 4.0 (1.1–22.4), *p* = 0.041) [22], indicating a potential risk in patients carrying this genotype.

**Table 1:** Genes and genetic variants related to statistically significant clinical response to biological therapies.

SNP	Study	*n*	Age in Years (Mean [SD] or Median [Range])	Ethnicity (Country)	HWE	Drug	Clinical Outcomes (Associated Allele, Genotype, or Haplotype)	HR/OR (95%CI) and/or *p*-Value	Observations *
***ADAM17***									
rs10929587	Laserna et al. (2023) [47]	CD: 131	36.4 [27.7–46.0]	Caucasian (Spain)	NR	IFX	TT: persistence of response	ORa: 0.2 (0.1–0.8), *p* = 0.021	1
***AHR***									
rs1077773	Burke et al. (2018) [33]	UC: 231	29.5 [NR]	Caucasian (NR)	Yes	Anti-TNF	G: persistence of response in UC	OR: 0.68, *p* = 0.004	2
	Yoon et al. (2017) [48]	CD: 314, UC: 145	Primary non-response: 29.6 [13.4], secondary non-response: 24.9 [13.8], durable response: 26.9 [13.4]	Caucasian > 84% (NR)	Yes	IFX, ADL, and CLZ for CD; IFX, ADL, and GOL for UC	G: primary non-response	OR: 0.61 (0.42–0.88), *p* = 0.008	
***ARFGAP2***									
rs3740691	Jezernik et al. (2023) [25]	CD: 102	27.4 [NR]	NR (Slovenia)	NR	ADL	GG: non-response	*p* = 1.24 × 10^−5^	3
***ATG16L1***									
rs12994997	Barber et al. (2016) [31]	CD: 427	25.7 [NR]	NR (USA)	Yes	IFX, ADL, and CLZ	G: persistence of response in CD	OR: 0.68, *p* = 0.05	
rs10210302	Koder et al. (2015) [17]	CD: 97	41.1 [11.9]	NR (Slovenia)	Yes	ADL	T: response	OR: 9.44 (2.49–35.83), *p* = 8.11 × 10^−4^	4
	Nuij et al. (2017) [49]	CD: 411, UC: 148, IC: 11	27 [5–79]	NR (Netherlands)	NR	IFX, ADL	T300A variant: response to ADL	OR: 2.4 (1.3–4.4), *p* = 0.004	
rs2241880	Dubinsky et al. (2010) [23]	IBD: 94	Age at diagnosis < 21	NR (USA)	Yes	IFX	T: non-response	OR: 2.8 (1.0–8.7), *p* = 0.048	5
	Zapata-Cobo (2023) [35]	CD: 240, UC: 93, IC: 7	11.2 [0.7–17.3]	NR (Spain)	Yes	IFX, ADL	AA: loss of response	OR: 0.551 (0.319–0.951) *p* = 0.030 (IBD) 0.320 (0.125–0.822) *p* = 0.012 (UC)	
rs7587051	Tang et al. (2020) [36]	CD: 189	23 [18–28]	NR (China)	Yes	IFX	GG: lower TL	OR: 1.65 (0.87–6.22), *p* = 0.015	
rs143063741	Tang et al. (2020) [36]	CD: 189	23 [18–28]	NR (China)	Yes	IFX	GG: lower TL	OR: 2.93 (1.31–5.40), *p* < 0.001	
***ATG5***									
rs9373839	Dezelak et al. (2016) [50]	CD: 79	26.6 [12.2]	NR (Slovenia)	NR	ADL	C: response	*p* < 0.01	
Haplotype rs9373839 rs510432	Dezelak et al. (2016) [50]	CD: 79	26.6 [12.2]	NR (Slovenia)	NR	ADL	CG: higher IBDQ scores	*p* < 0.01	
rs3740691	Repnik et al. (2019) A [51]	CD: 102	NR	NR	NR	ADL	A: non-response after four weeks of treatment	*p* = 1.20 × 10^−5^	
***ATXN2***									
rs653178	Burke et al. (2018) [33]	UC: 231	29.5 [NR]	Caucasian (NR)	Yes	Anti-TNF	C: non-response	OR: 1.78, *p* = 0.049	2
***ATXN2L***									
rs35725751	Barber et al. (2016) [31]	CD: 427	25.7 [NR]	NR (USA)	Yes	IFX, ADL, and CLZ	T: persistence of response in CD	OR: 0.45, *p* = 0.01	
***BRWD1***									
rs2836878	Dubinsky et al. (2010) [23]	IBD: 94	Age at diagnosis < 21	NR (USA)	Yes	IFX	GG: non-response	OR: 2.6 (1.0–6.5), *p* = 0.03	5
***C1orf106***									
rs442905	Tang et al. (2020) [36]	CD: 189	23 [18–28]	NR (China)	Yes	IFX	GA: lower TL	*p* = 0.046	
rs59457695	Tang et al. (2020) [36]	CD: 189	23 [18–28]	NR (China)	Yes	IFX	CC: higher TL	*p* = 0.022	
	Tang et al. (2020) [36]	CD: 190	23 [18–28]	NR (China)	Yes	IFX	T: response	OR: 1.47 (1.33–1.63) *p* = 0.034	
rs61740234	Zhang et al. (2021) [24]	CD: 206	Responders: 23.0 [18.0–27.6], non-responders: 23.5 [16.8–33.5]	NR (China)	Yes	IFX	CC + TT: non-response	OR: 4.49 (1.31–15.32), *p* = 0.010	6
***C11orf9***									
rs174537	Zhu et al. (2017) [52]	CD: 485, UC: 172	NR	NR	NR	Anti-TNF	Non-response	OR: 2.6, *p* = 0.049	
***C11orf30***									
rs7927894	Koder et al. (2015) [17]	CD: 97	41.1 [11.9]	NR (Slovenia)	Yes	ADL	CC: response	OR: *p* = 5.83 × 10^−3^	4
***CASP9***									
rs4645983	Hlavaty et al. (2005) [28]	CD: 287	Luminal: 24.9 [11.1], fistulizing: 26.2 [12.0]	NR (Belgium)	Yes	IFX	CT: Response	OR: 1.50 (1.34–1.68), *p* = 0.04	
	Koder et al. (2015) [17]	CD: 97	41.1 [11.9]	NR (Slovenia)	Yes	ADL	T: response	*p* = 6.91 × 10^−3^	4
***CCDC88B***									
rs61886887	Zhang et al. (2021) [24]	CD: 206	Responders: 23.0 [18.0–27.6], non-responders: 23.5 [16.8–33.5]	NR (China)	Yes	IFX	T: non-response	OR: 0.08 (0.01–0.61), *p* = 0.002	6
***CCL2, CCL7***									
rs3091315	Burke et al. (2018) [33]	UC: 231	29.5 [NR]	Caucasian (NR)	Yes	Anti-TNF	G: persistence of response in UC	OR: 0.63, *p* = 0.024	2
***CCNY***									
rs12777960	Koder et al. (2015) [17]	CD: 97	41.1 [11.9]	NR (Slovenia)	Yes	ADL	CC: response	*p* = 6.69 × 10^−3^	4
***CD14***									
rs2569190	Bank et al. (2014) [18]	IBD: 738, CD: 482, UC: 256	NR [04–81]	NR (Denmark)	NR	Anti-TNF	A: non-response in UC	OR: 0.54 (0.3–0.98), *p* = 0.04	
	Jezernik et al. (2023) [25]	CD: 102	27.4 [NR]	NR (Slovenia)	NR	ADL	G: non-response at week 30	*p* = 2.60 × 10^−2^	3
	Salvador-Martín et al. (2019) A [32]	IBD: 210, CD: 147, UC: 63	NR (<18 years)	NR (Spain)	NR	IFX, ADL	A: lower TL	*p* = 0.044	
***CD28***									
rs3116494	Yoon et al. (2017) [48]	CD: 314, UC: 145	Primary non-response: 29.6 [13.4], secondary non-response: 24.9 [13.8], durable response: 26.9 [13.4]	Caucasian > 84% (NR)	Yes	IFX, ADL, and CLZ for CD; IFX, ADL, and GOL for UC	G: persistence of response	OR: 1.46 (1.20–1.79), *p* = 0.0002	
***CDKAL1***									
rs6908425	Dubinsky et al. (2010) [23]	IBD: 94	Age at diagnosis < 21	NR (USA)	Yes	IFX	T: non-response	OR: 2.1 (1.0–4.3), *p* = 0.049	5
	Jezernik et al. (2023) [25]	CD: 102	27.4 [NR]	NR (Slovenia)	NR	ADL	T: non-response	*p* = 1.73 × 10^−2^	3
	Zapata-Cobo (2023) [35]	CD: 240, UC: 93, IC: 7	11.2 [0.7–17.3]	NR (Spain)	Yes	IFX, ADL	CC: loss of response in CD	OR: 2.410 (1.107–5.250) *p* = 0.022	
***CD96***									
rs9828223	Aterido et al. (2019) [53]	Discovery cohort: 62, CD replication cohort: 88 CD	Discovery cohort: 39 [12.2], replication cohort: 37 [12.85]	Caucasian (Spain)	Yes	ADL	A: ADA development and non-response	ADA development: OR: 15.87 (4.38–57.49), *p* = 1.83 × 10^−9^ non-response: OR: 1.77 (1.09–5.02), *p* = 0.019	7
***CHSY2, HINT1***									
rs10051722	Zhu et al. (2017) [52]	CD: 485, UC: 172	NR	NR	NR	Anti-TNF	Non-response	OR: 0.2, *p* = 0.03	
	Zhu et al. (2017) [52]	CD: 485, UC: 172	NR	NR	NR	Anti-TNF	ADA development	*p* = 0.014	
***CRP***									
rs1130864	Dezelak et al. (2016) [50]	CD: 79	26.6 [12.2]	NR (Slovenia)	NR	ADL	C: higher IBDQ scores	*p* < 0.01	
***CRTC3***									
rs7495132	Zhu et al. (2017) [52]	CD: 485, UC: 172	NR	NR	NR	Anti-TNF	ADA development	*p* = 0.026	
***CNTF, LPXN***									
rs11229555	Barber et al. (2016) [31]	CD: 427	25.7 [NR]	NR (USA)	Yes	IFX, ADL, and CLZ	T: persistence of response in CD	OR: 0.58, *p* < 0.01	
***CXCL12***									
rs10508884	Zapata-Cobo (2023) [35]	CD: 240, UC: 93, IC: 7	11.2 [0.7–17.3]	NR (Spain)	Yes	IFX, ADL	C: loss of response	OR: 0.269 (0.066–1.096) *p* = 0.049	
***CYP1A2***									
rs762551	Wang et al. (2021) [54]	IBD: 51	39 [17.5]	NR (European countries)	Yes	IFX, ADL	1A/1F + 1F/1F: non-response	*p* = 0.04	8
***DAXX***									
rs2239839	Yoon et al. (2017) [48]	CD: 314, UC: 145	Primary non-response: 29.6 [13.4], secondary non-response: 24.9 [13.8], durable response: 26.9 [13.4]	Caucasian > 84% (NR)	Yes	IFX, ADL, and CLZ for CD; IFX, ADL, and GOL for UC	A: non-response	OR: 2.08 (1.45–2.99), *p* = 7.37 × 10^−5^	
***DENND1B***									
rs2488397	Yoon et al. (2017) [48]	CD: 314, UC: 145	Primary non-response: 29.6 [13.4], secondary non-response: 24.9 [13.8], durable response: 26.9 [13.4]	Caucasian > 84% (NR)	Yes	IFX, ADL, and CLZ for CD; IFX, ADL, and GOL for UC	C: non-response	OR: 1.85 (1.26–2.72), *p* = 0.002	
imm_1_195969024	Zhu et al. (2017) [52]	CD: 485, UC: 172	NR	NR	NR	Anti-TNF	Non-response	OR: 2.8, *p* = 0.03	
***DNMT3B***									
rs6087990	Barber et al. (2016) [31]	CD: 427	25.7 [NR]	NR (USA)	Yes	IFX, ADL, and CLZ	C: persistence of response in CD	OR: 1.64, *p* = 0.01	
***DTNBP1***									
rs10456777	Yoon et al. (2017) [48]	CD: 314, UC: 145	Primary non-response: 29.6 [13.4], secondary non-response: 24.9 [13.8], durable response: 26.9 [13.4]	Caucasian > 84% (NR)	Yes	IFX, ADL, and CLZ for CD; IFX, ADL, and GOL for UC	C: non-response	OR: 3.63 (2.09–6.32), *p* = 5.15 × 10^−6^	
***ELOVL7***									
rs9291695	Gorenjak et al. (2021) [55]	CD: 84	Responders: 27.2 [12.3], non-responders: 29.2 [12.0]	Caucasian (Slovenia)	NR	ADL	A: non-response	OR: 0.07 (0.02–0.32), *p* < 0.01	
rs78620886	Gorenjak et al. (2021) [55]	CD: 84	Responders: 27.2 [12.3], non-responders: 29.2 [12.0]	Caucasian (Slovenia)	NR	ADL	A: non-response	OR: 0.05 (0.01–0.27), *p* < 0.01	
***ERGIC1***									
rs564349	Zhu et al. (2017) [52]	CD: 485, UC: 172	NR	NR	NR	Anti-TNF	Non-response	OR: 2.9, *p* = 0.021	
***FAS***									
rs7896789	Lykowska-Szuber et al. (2023) [26]	CD: 196	30.65 [10.58]	NR (Poland)	No	IFX, ADL	CC: non-response	OR: 15.22, *p* = 0.003	
	Walczak et al. (2020) [27]	CD: 107	28 [10.64]	NR (Poland)	Yes	IFX, ADL	C: non-response	OR: 3.63 (1.261–10.425), *p* = 0.03	9
***FASLG***									
rs763110	Hlavaty et al. (2005) [28]	CD: 287	Luminal: 24.9 [11.1], fistulizing: 26.2 [12.0]	NR (Belgium)	Yes	IFX	CT: non-response	OR: 0.11 (0.08–0.56), *p* = 0.002	
	Netz et al. (2017) [29]	CD: 121	41.6 [95% CI: 39.2–44.0]	Caucasian 93% (USA)	Yes	Anti-TNF	CC: non-response	OR: 4.30 (1.45–12.80), *p* = 0.009	
	Salvador-Martín et al. (2019) A [32]	IBD: 210, CD: 147, UC: 63	NR (<18 years)	NR (Spain)	NR	IFX, ADL	C: lower TL	*p* = 0.032	
	Steenholdt et al. (2012) [22]	CD: 104	Primary non-response: 22 [19–42], loss of response: 24 [20–38], maintained remission: 25 [20–32]	Caucasian (NR)	Yes	IFX	T: more risk of adverse events with IFX	OR: 4.0 (1.1–22.4), *p* = 0.041	
***FCGR3A***									
rs396991	Curci et al. (2021) [38]	CD: 50, UC: 26	14.3 [12.3–16.3]	NR (Italy)	Yes	IFX	C: non-response, lower TL, and ADA development	W14:AC:OR: 6.76 (1.89–24.19) (*p* = 0.003) CC:OR: 5.43 (1.22–24.07) (*p* = 0.026) W22:AC:OR: 8.89 (2.52–31.41) (*p* = 0.001) CC:OR: 4.39 (1.05–18.35) (*p* = 0.042) W52:AC:OR: 6.58 (1.91–23.17) (*p* = 0.003) CC:OR: 2.25 (0.61–10.11) (*p* = 0.198)	
	Zhu et al. (2023) [39]	CD: 104	29 [23–33]	NR (China)	Yes	IFX	C: development of ADA	OR: 2.94 (1.24–6.96), *p* = 0.01	
	Romero-Cara et al. (2018) [40]	IBD: 103, CD: 80, UC: 23	43.7 [13.6]	NR (Spain)	Yes	IFX, ADL	VV: ADA development	*p* = 0.004	
	Louis et al. (2004) [56]	CD: 200	34 [25–47]	NR (Belgium)	NR	IFX	VV: response	RR: 1.19 (0.99–1.43), *p* = 0.15	
	Louis et al. (2006) [57]	CD: 344	35 [18–76]	Caucasian (Belgium)	Yes	IFX	VV: response	*p* = 0.043	
	Moroi et al. (2013) [58]	CD: 102	30 [24–37]	NR (Japan)	NR	IFX	VV: response	OR: 9.09 (2.26–36.63), *p* = 0.000296	
	Ternant et al. (2014) [59]	CD: 111	31 [25–39]	NR (France and Belgium)	NR	IFX	VV: increased IFX elimination	*p* = 0.0028	
	Ternant et al. (2014) [59]	CD: 111	31 [25–39]	NR (France and Belgium)	NR	IFX	VV: loss of response after IFX discontinuation	HR 1.7 (1.1–2.6), *p* = 0.013	
rs111504845	Zhang et al. (2021) [24]	CD: 206	Responders: 23.0 [18.0–27.6], non-responders: 23.5 [16.8–33.5]	NR (China)	Yes	IFX	G: non-response	OR: 2.50 (1.00–6.33), *p* = 0.047	6
rs7539036	Walczak et al. (2020) [27]	CD: 107	28 [10.64]	NR (Poland)	Yes	IFX, ADL	T: non-response	OR: 3.63 (1.261–10.425), *p* = 0.01	9
rs6672453	Walczak et al. (2020) [27]	CD: 107	28 [10.64]	NR (Poland)	Yes	IFX, ADL	T: non-response	OR: 3.63 (1.261–10.425), *p* = 0.01	9
rs373184583	Walczak et al. (2020) [27]	CD: 107	28 [10.64]	NR (Poland)	Yes	IFX, ADL	T: non-response	OR: 3.63 (1.261–10.425), *p* = 0.01	9
rs12128686	Walczak et al. (2020) [27]	CD: 107	28 [10.64]	NR (Poland)	Yes	IFX, ADL	G: non-response	OR: 3.63 (1.261–10.425), *p* = 0.01	9
***FGCR3B***									
FCGR3B (FcgammaRIIIB-NA1/NA2)	Tomita et al. (2010) [60]	CD: 41	30 [18–54]	NR (Japan)	NR	IFX	NA1/NA1: response	*p* = 0.042	
***FCGR1A***									
FCGR1A	Salvador-Martín et al. (2021) A [61]	IBD: 38, CD: 30, UC: 8	10.5 [0.7–17]	NR (Spain)	NR	IFX, ADL	Non-response	*p* = 0.006	
***FCGR1B***									
FCGR1B	Salvador-Martín et al. (2021) A [61]	IBD: 38, CD: 30, UC: 8	10.5 [0.7–17]	NR (Spain)	NR	IFX, ADL	Non-response	*p* = 0.005	
***FCGRT***									
VNTR polymorphism	Billiet et al. (2016) [62]	IFX group: CD 239, UC 146 ADL group: CD 100, UC 39	IFX group: 37.3 [26.4–48.6], ADL group: 41.8 [30–53.4]	NR (Belgium)	Yes	IFX, ADL	2/3: lower AUC	IFX *p* = 0.03 ADL *p* = 0.005	
	Choi et al. (2023) [63]	IBD: 156	WT: 46 [18–74] variant carrier: 49 [20–72]	NR (Canada)	Yes	IFX	2/3 or 2/2: lower TL	*p* < 0.01	
***FIBP***									
rs568617	Burke et al. (2018) [33]	UC: 231	29.5 [NR]	Caucasian (NR)	Yes	Anti-TNF	T: non-response	OR: 0.39, *p* = 0.042	2
***G0S2***									
rs4844486, rs1473683	Medrano et al. (2015) [64]	CD: 350	Responders: 39.6 [0.7], non-responders: 41.9 [1.5]	NR (Spain)	Yes	IFX	AT haplotype: non-response	OR: 6.31 (0.82–47.89), *p* = 0.025	
***GBP1***									
GBP1	Salvador-Martín et al. (2021) A [61]	IBD: 38, CD: 30, UC: 8	10.5 [0.7–17]	NR (Spain)	NR	IFX, ADL	Non-response	*p* = 0.006	
***GJB3***									
rs145751680	Jung et al. (2019) [65]	CD: 104, UC: 31	Primary responders: 25.85 [10.88], non-responders: 24.52 [10.80]	Asian (Korea)	Yes	IFX	c.250G > A: primary non-response	*p* = 2.24 × 10^−6^	
***GLIS3***									
rs1330307	Burke et al. (2018) [33]	UC: 231	29.5 [NR]	Caucasian (NR)	Yes	Anti-TNF	C: non-response	OR: 0.23, *p* < 0.01	2
***GNA12***									
rs1182188	Barber et al. (2016) [31]	CD: 427	25.7 [NR]	NR (USA)	Yes	IFX, ADL, and CLZ	C: non-response in CD	OR: 1.86, *p* = 0.01	
***GSN***									
rs55689715	Barber et al. (2016) [31]	CD: 427	25.7 [NR]	NR (USA)	Yes	IFX, ADL, and CLZ	C: persistence of response in CD	OR: 3.32, *p* < 0.01	
***HFE***									
rs2071303	Repnik et al. (2016) [66]	CD: 68	NR	NR (Slovenia)	NR	ADL	G: response after 20 weeks	OR: 3.542 (1.355–9.258), *p* = 0.008	
***HHEX, EXOC6***									
rs12778642	Zhu et al. (2017) [52]	CD: 485, UC: 172	NR	NR	NR	Anti-TNF	ADA development	*p* = 0.004	
rs9264942	Zhu et al. (2017) [52]	CD: 485, UC: 172	NR	NR	NR	Anti-TNF	Non-response	OR: 3.4, *p* = 0.041	
***HLA-DQA1***									
rs2097432 (*05)	Guardiola et al. (2020) [67]	CD: 53	NR	NR (Spain)	NR	ADL	*05: loss of response	HR 2.1 (95%CI 1.1–4.3), *p* = 0.04	
	Suris-Marin et al. (2021) [68]	CD: 65, UC: 34	NR	NR (Spain)	NR	IFX	*05: 39% failure		
	Cheli et al. (2023) [46]	CD: 56, UC: 23	13 [2.5]	NR (Italy)	Yes	IFX, ADL	No difference between genotypes		
	Fuentes-Valenzuela et al. (2023) [69]	CD: 90, UC: 32	Non-carriers: 40.3 [25.3–49.7], carriers 37.6 [25.3–47.1]	NR (Spain)	NR	IFX, ADL	*05: netter persistence	HR = 0.32 (0.14–0.71) *p* = 0.01	
	Ioannou et al. (2021) [42]	IBD: 610, CD: 396, UC: 200, IC: 14	Carriers: 25 [7–68], non-carriers: 22.5 [3–68]	Hispanic (NR)	NR	IFX, ADL, UST, VDZ	*05: development of ADA	ORa: 4.47 (1.18–16.49), *p* = 0.02	
	Salvador-Martín et al. (2023) [70]	IBD: 340, CD: 240, UC: 93, IC: 7	27.2 [10.8–76.7]	NR (Spain)	No	IFX, ADL	TT: persistence of response	HR: 1.770 (1.100–2.848), *p* = 0.019	
	Sazonovs et al. (2020) [43]	CD: 1240	IFX: 24.4 [16.4–35.9], ADL: 29.5 [21.8–41.8]	NR (European countries)	Yes	IFX, ADL	*05: ADA development	HR 1.90 (1.60–2.25), *p* = 5.88 × 10^13^	
	Spencer et al. (2022) [71]	IBD: 151	NR	NR (USA)	NR	IFX	Pre-therapy clearance > 0.30 L/day and *05: response	HR: 0.48 (0.28–0.83), *p* < 0.01	
	Spencer et al. (2024) [44]	IBD: 415	27 [15–43]	NR	NR	IFX, ADL	*05: ADA development	OR: 1.9 (1.4–2.8), *p* < 0.001	
	Wilson. A (2019) [45]	CD: 152, UC: 110	39.66 [18–79]	NR (Canada)	Yes	IFX	*05: ADA development and loss of response	Loss of response: HRa: 2.34 (1.41–3.88), *p* = 0.001. Discontinuation: HRa: 2.27 (1.46–3.43), *p* = 2.53 × 10^−4^	
	Zhu et al. (2023) [39]	CD: 104	29 [23–33]	NR (China)	Yes	IFX	G: development of ADA	OR: 2.94 (1.19–7.30), *p* = 0.02	
rs74291249	Choi et al. (2023) [63]	IBD: 156	NR [18–74]	NR (Canada)	Yes	IFX	A: lower TL	*p* < 0.01	
rs2395185	Dubinsky et al. (2010) [23]	IBD: 94	Age at diagnosis < 21	NR (USA)	Yes	IFX	GG: primary non-response	OR: 2.9 (1.3–6.4), *p* = 0.007	5
***HLA-DRB9***									
rs2395185	Salvador-Martín et al. (2023) [70]	IBD: 340, CD: 240, UC: 93, IC: 7	27.2 [10.8–76.7]	NR (Spain)	Yes	IFX, ADL	T: persistence response	HR: 0.599 (0.368–0.975), *p* = 0.039	
***IBD5***									
IGR2060a1 and IGR3081a1	Urcelay et al. (2005) [72]	CD: 40	Responders: 35 [21–66], non-responders: 40 [17–68]	Caucasian (Spain)	NR	IFX	The homozygous mutant 5q31 genotype: non-response	RR = 3.88 (1.18–12.0), *p* < 0.05	
***ICOSLG***									
rs762421	Dubinsky et al. (2010) [23]	IBD: 94	Age at diagnosis < 21	NR (USA)	Yes	IFX	AA: primary non-response	OR: 2.6 (1.0–7.0), *p* = 0.045	5
	Jezernik et al. (2023) [25]	CD: 102	27.4 [NR]	NR (Slovenia)	NR	ADL	A: non-response	*p* = 0.00211	2
***IFIH1***									
rs1990760	Yoon et al. (2017) [48]	CD: 314, UC: 144	Primary non-response: 29.6 [13.4], secondary non-response: 24.9 [13.8], durable response: 26.9 [13.4]	Caucasian > 84% (NR)	Yes	IFX, ADL, and CLZ for CD; IFX, ADL, and GOL for UC	G: persistence of response	HR: 0.79 (0.65–0.95), *p* = 0.01	
rs2111485	Yoon et al. (2017) [48]	CD: 314, UC: 145	Primary non-response: 29.6 [13.4], secondary non-response: 24.9 [13.8], durable response: 26.9 [13.4]	Caucasian > 84% (NR)	Yes	IFX, ADL, and CLZ for CD; IFX, ADL, and GOL for UC	A: persistence of response	HR: 0.74 (0.61–0.89), *p* = 0.002	
***IFNG***									
rs2430561	Bank et al. (2014) [18]	IBD: 738, CD: 482, UC: 256	NR [04–81]	NR (Denmark)	NR	Anti-TNF	A: response in combined IBD and UC	OR: 1.66 (1.05–2.62), *p* = 0.03 (IBD)	10
	Jezernik et al. (2023) [25]	CD: 102	27.4 [NR]	NR (Slovenia)	NR	ADL	AA: non-response	*p* = 0.0373	3
***IFNGR1***									
rs2234711	Bank et al. (2018) [73]	CD: 482, UC: 256	NR [04–77]	Caucasian (Denmark)	NR	Anti-TNF	TC: non-response in UC TC: non-response in combined IBD	OR: 0.29 (0.11–0.78), *p* = 0.01 (UC) OR: 0.57 (0.34–0.96), *p* = 0.04 (IBD)	11
***IFNGR2***									
rs8126756	Bank et al. (2018) [73]	CD: 482, UC: 256	NR [04–77]	Caucasian (Denmark)	NR	Anti-TNF	CC: non-response in CD	OR: 0.09 (0.01–0.65), *p* = 0.02	11
rs2284553	Burke et al. (2018) [33]	UC: 231	29.5 [NR]	Caucasian (NR)	Yes	Anti-TNF	A: non-response	OR: 1.80, *p* = 0.037	2
***IL6***									
rs10499563	Bank et al. (2014) [18]	IBD: 738, CD: 482, UC: 256	NR [04–81]	NR (Denmark)	NR	Anti-TNF	C: response in combined IBD	OR: 2.26 1.18–4.32, *p* = 0.01	10
	Salvador-Martín et al. (2019) A [32]	IBD: 210, CD: 147, UC: 63	NR (<18 years)	NR (Spain)	NR	IFX, ADL	C: greater persistence in CD	HR: 0.2 (0.05–1), *p* = 0.05	
	Salvador-Martín et al. (2019) B [34]	CD: 132	27.4 [10.8–76.7]	NR (Spain)	Yes	IFX	CC: higher TL	OR: 21.43 (2.34–196.43), *p* = 0.007	
	Salvador-Martín et al. (2020) [19]	IBD: 209, CD: 147, UC: 62	10.6 [0.7–17.3]	NR (Spain)	No	IFX, ADL	TT: response in CD	HR: 0.210 (0.047–0.947), *p* = 0.042	
***IL10***									
rs1800872	Salvador-Martín et al. (2019) A [32]	IBD: 210, CD: 147, UC: 63	NR (<18 years)	NR (Spain)	NR	IFX, ADL	C: greater persistence in CD	HR: 4.2 (1–17), *p* = 0.047	
	Salvador-Martín et al. (2019) B [34]	CD: 132	27.4 [10.8–76.7]	NR (Spain)	Yes	IFX	A: higher TL	CC vs. CA:OR: 9.48 (1.05–85.71), *p* = 0.045; CC vs. AA:OR: 20.84 (1.41–307.79), *p* = 0.027	
	Salvador-Martín et al. (2020) [19]	IBD: 209, CD: 147, UC: 62	10.6 [0.7–17.3]	NR (Spain)	No	IFX, ADL	CC: response in CD	HR: 4.749 (1.156–19.517), *p* = 0.031	
rs3024505	Salvador-Martín et al. (2019) B [34]	CD: 132	27.4 [10.8–76.7]	NR (Spain)	Yes	IFX	T: lower TL	OR: 0.33 (0.12–0.92), *p* = 0.033	
rs1800896	Jezernik et al. (2023) [25]	CD: 102	27.4 [NR]	NR (Slovenia)	NR	ADL	C: non-response	*p* = 0.0122	2
rs3021094	Tang et al. (2020) [36]	CD: 189	23 [18–28]	NR (China)	Yes	IFX	GG: higher TL	*p* = 0.013	
***IL11***									
rs1126760, rs1042506	Medrano et al. (2015) [64]	CD: 350	Responders: 39.6 [0.7], non-responders: 41.9 [1.5]	NR (Spain)	Yes	IFX	CT haplotype: non-response	OR: 2.76 (1.39–5.44), *p* = 0.025	
***IL13***									
rs1295686	Koder et al. (2015) [17]	CD: 97	41.1 [11.9]	NR (Slovenia)	Yes	ADL	AA: response	*p* = 0.00607	4
***IL17A***									
rs2275913	Bank et al. (2014) [18]	IBD: 738, CD: 482, UC: 256	NR [04–81]	NR (Denmark)	NR	Anti-TNF	A: non-response in combined IBD and UC	OR: 0.42 (0.18–1.00), *p* = 0.05 (UC)	10
	Jezernik et al. (2023) [25]	CD: 102	27.4 [NR]	NR (Slovenia)	NR	ADL	GG: response	*p* = 9.73 × 10^−3^	3
	Repnik et al. (2019) B [74]	CD: 102	NR	NR (Slovenia)	NR	ADL	Response	*p* = 0.006	12
	Salvador-Martín et al. (2019) A [32]	IBD: 210, CD: 147, UC: 63	NR (<18 years)	NR (Spain)	NR	IFX, ADL	G: greater persistence	HR: 0.3 (0.1–0.9), *p* = 0.035	
	Salvador-Martín et al. (2020) [19]	IBD: 209, CD: 147, UC: 62	10.6 [0.7–17.3]	NR (Spain)	Yes	IFX, ADL	AA: response in CD	HR: 0.320 (0.111–0.920), *p* = 0.034	
rs2241046	Zhang et al. (2021) [24]	CD: 206	Responders: 23.0 [18.0–27.6], non-responders: 23.5 [16.8–33.5]	NR (China)	Yes	IFX	T: primary non-response	OR: 0.17 (0.04–0.80), *p* = 0.012	6
***IL17F***									
rs766748	Urabe S. et al. (2015) [75]	CD: 103	Responders: 35.4 [12.9], non-responders: 37.8 [10.3]	NR (Japan)	YES	IFX	GG: response after 1 year of treatment	OR: 5.123 (1.261–27.77), *p* = 0.0213	
***IL1R***									
rs2041747	Lykowska-Szuber et al. (2023) [26]	CD: 196	30.65 [10.58]	NR (Poland)	Yes	IFX, ADL	G: non-response to induction therapy	OR: 3.72, *p* = 0.009	
	Walczak et al. (2020) [27]	CD: 107	28 [10.64]	NR (Poland)	Yes	IFX, ADL	A: non-response	OR: 5.29 (1.178–23.708), *p* = 0.02	9
***IL1RN***									
rs4251961	Bank et al. (2014) [18]	IBD: 738, CD: 482, UC: 256	NR [04–81]	NR (Denmark)	NR	Anti-TNF	C: non-response in UC	OR: 0.42 (0.18–0.98), *p* = 0.04	10
	Bank et al. (2019) [20]	IBD: 1045, CD: 587, UC: 458	NR [3–84]	NR (Denmark)	NR	IFX, ADL, GOL	C: non-response in IBD	OR: 0.81 (0.66–1.00), *p* = 0.049	13
	Jezernik et al. (2023) [25]	CD: 102	27.4 [NR]	NR (Slovenia)	NR	ADL	CC: non-response	*p* = 0.0393	3
rs3213448	Tang et al. (2020) [36]	CD: 189	23 [18–28]	NR (China)	Yes	IFX	GA: higher TL	*p* = 0.011	
rs3213449	Tang et al. (2020) [36]	CD: 190	23 [18–28]	NR (China)	Yes	IFX	G: CRP level at 14 weeks	*p* = 0.005	
	Zhang et al. (2021) [24]	CD: 206	Responders: 23.0 [18.0–27.6], non-responders: 23.5 [16.8–33.5]	NR (China)	Yes	IFX	TT + CC: non-response	OR: 2.18 (1.05–4.56), *p* = 0.035	6
***IL1B***									
rs4848306	Bank et al. (2014) [18]	IBD: 738, CD: 482, UC: 256	NR [04–81]	NR (Denmark)	NR	Anti-TNF	A: response in combined IBD and UC	OR: 1.85 (1.05–3.27), *p* = 0.03 (IBD)	10
	Jezernik et al. (2023) [25]	CD: 102	27.4 [NR]	NR (Slovenia)	NR	ADL	GG: non-response	*p* = 0.0223	3
rs1143634	Guardiola et al. (2016) [76]	IBD: 67	NR	NR (Spain)	NR	IFX	A: lower TL		Abstract
	Lacruz et al. (2013) [30]	CD: 29, UC: 18	CD: 30.8 [13.6], UC: 39.7 [14.9]	Caucasian (Spain)	Yes	IFX	C: non-response in CD	*p* = 0.027	
	Lykowska-Szuber et al. (2023) [26]	CD: 196	30.65 [10.58]	NR (Poland)	Yes	IFX, ADL	No difference between genotypes		
	Santacana et al. (2016) [77]	IBD: 67, CD: 44, UC: 23	NR	NR (Spain)	NR	IFX	CC: lower median TL, TL/D and AUC	TL: *p* = 0.013; TL/D: *p* = 0.019; AUC: *p* = 0.023	C allele developed antibodies toward IFX
	Walczak et al. (2020) [27]	CD: 107	28 [10.64]	NR (Poland)	Yes	IFX, ADL	T: non-response	OR: 2.59 (1.093–6.113), *p* = 0.03	9
rs1071676	Walczak et al. (2020) [27]	CD: 107	28 [10.64]	NR (Poland)	Yes	IFX, ADL	C: non-response	OR: 2.59 (1.093–6.113), *p* = 0.03	9
rs1143639	Walczak et al. (2020) [27]	CD: 107	28 [10.64]	NR (Poland)	Yes	IFX, ADL	A: non-response	OR: 2.59 (1.093–6.113), *p* = 0.03	9
rs1143637	Walczak et al. (2020) [27]	CD: 107	28 [10.64]	NR (Poland)	Yes	IFX, ADL	A: non-response	OR: 2.59 (1.093–6.113), *p* = 0.03	9
***IL2RA***									
rs12722515	Barber et al. (2016) [31]	CD: 427	25.7 [NR]	NR (USA)	Yes	IFX, ADL and CLZ	A: persistence of response in CD	OR: 3.00, *p* < 0.01	
***IL12B***									
rs32112217	Bank et al. (2017) [73]	CD: 482, UC: 256	NR [04–77]	Caucasian (Denmark)	NR	Anti-TNF	GC: non-response in UC	OR: 0.35 (0.13–0.92), *p* = 0.03	11
***IL18***									
rs187238	Bank et al. (2019) [20]	IBD: 1045, CD: 587, UC: 458	NR [3–84]	NR (Denmark)	NR	IFX, ADL, GOL	C: response in CD	OR: 1.35 (1.00–1.82), *p* = 0.047	13
rs1946518	Bank et al. (2019) [20]	IBD: 1045, CD: 587, UC: 458	NR [3–84]	NR (Denmark)	NR	IFX, ADL, GOL	T: response in IBD	OR: 1.24 (1.01–1.53), *p* = 0.04	13
***IL18RAP***									
rs6708413	Barber et al. (2016) [31]	CD: 427	25.7 [NR]	NR (USA)	Yes	IFX, ADL, and CLZ	G: non-response	OR: 1.71, *p* = 0.04	
***IL23R***									
rs10489629	Laserna et al. (2023) [47]	CD: 131	36.4 [27.7–46.0]	Caucasian (Spain)	NR	IFX	TT: persistence of response	*p* = 0.001	1
***IL27***									
rs8049439	Koder et al. (2015) [17]	CD: 97	41.1 [11.9]	NR (Slovenia)	Yes	ADL	T: response	OR: 9.67 (1.65–56.55), *p* = 0.00971	4
***IPMK***									
rs2790216	Burke et al. (2018) [33]	UC: 231	29.5 [NR]	Caucasian (NR)	Yes	Anti-TNF	A: better persistence of response in UC	OR: 1.56, *p* = 0.042	2
***IRF1-AS1* (IncRNA)**									
rs2188962	Dubinsky et al. (2010) [23]	IBD: 94	Age at diagnosis < 21	NR (USA)	Yes	IFX	CC: non-response	OR: 2.3 (1.1–4.7), *p* = 0.028	5
	Zapata-Cobo (2023) [35]	CD: 240, UC: 93, IC: 7	11.2 [0.7–17.3]	NR (Spain)	Yes	IFX, ADL	T: loss of response in UC	OR: 3.155 (1.100–9.047), *p* = 0.024	
***IRGM***									
rs13361189	Jezernik et al. (2023) [25]	CD: 102	27.4 [NR]	NR (Slovenia)	NR	ADL	C: non-response	*p* = 0.0445	3
	Nuij et al. (2017) [49]	CD: 411, UC: 148, IC: 11	27 [5–79]	NR (Netherlands)	NR	IFX, ADL	C: non-response to IFX in UC; C: better response to ADL	IFX: 12.2 (1.2–78.8), *p* = 0.009 ADL: 0.58 (0.36–0.92), *p* = 0.021	
***ITLN1***									
rs2274910	Jezernik et al. (2023) [25]	CD: 102	27.4 [NR]	NR (Slovenia)	NR	ADL	T: non-response	*p* = 0.0479	3
***JAK2***									
rs12343867	Bank et al. (2018) [73]	CD: 482, UC: 256	NR [04–77]	Caucasian (Denmark)	NR	Anti-TNF	CC: non-response in UC	OR: 0.17 (0.03–0.85), *p* = 0.03	11
	Bank et al. (2019) [20]	IBD: 1045, CD: 587, UC: 458	NR [3–84]	Caucasian (Denmark)	NR	IFX, ADL, GOL	C: response in CD and IBD	OR: 1.35 (1.02–1.78), *p* = 0.03 (CD) OR: 1.24 (1.01–1.53), *p* = 0.04 (IBD)	13
***KLHL1***									
rs9572250	Wang et al. (2019) [78]	IBD: 474, CD: 359, UC: 99, IC: 16	Responders: 29.8 [13.4], non-responders: 26.4 [12.1]	NR (European countries)	Yes	IFX, ADL	G: non-response	ORa 3.00 (1.84–4.89), *p* = 3.19 × 10^−6^	14
***KRT4***									
rs7956809	Barber et al. (2016) [31]	CD: 427	25.7 [NR]	NR (USA)	Yes	IFX, ADL, and CLZ	G: non-response in CD	OR: 3.20, *p* < 0.01	
***LINC02943***									
rs2836866	Yoon et al. (2017) [48]	CD: 314, UC: 145	Primary non-response: 29.6 [13.4], secondary non-response: 24.9 [13.8], durable response: 26.9 [13.4]	Caucasian > 84% (NR)	Yes	IFX, ADL, and CLZ for CD; IFX, ADL, and GOL for UC	G: persistence of response	HR: 1.54 (1.24–1.91), *p* = 7.86 × 10^−5^	
***LSP1***									
rs907611	Burke et al. (2018) [33]	UC: 231	29.5 [NR]	Caucasian (NR)	Yes	Anti-TNF	A: better persistence of response in UC	OR: 1.54, *p* = 0.048	2
***LTA***									
Haplotype NcoI-TNFc-aa13L-aa26	Ozeki et al. (2006) [79]	CD: 98	NR	NR (Japan)	Yes	IFX	Homozygosity for an LTA haplotype (LTA 1-1-1-1): non-response	NR	
	Taylor et al. (2001) [80]	CD: 75	NR	NR (USA)	No	IFX	Homozygosity for an LTA haplotype (LTA 1-1-1-1): non-response	*p* = 0.007	
***LTB***									
rs769177	Perera et al. (2010) [81]	IBD: 105	NR	Caucasian (NR)	NR	Anti-TNF	Non-response	OR: 5.7, *p* = 0.004	
***LTF***									
rs762787	Wang et al. (2019) [78]	IBD: 474, CD: 359, UC: 99, IC: 16	Responders: 29.8 [13.4], non-responders: 26.4 [12.1]	NR (European countries)	Yes	IFX, ADL	T: non-response	ORa 4.27 (2.16–8.48), *p* = 6.74 × 10^−6^	14
***LUZP2***									
rs1915063	Yoon et al. (2017) [48]	CD: 314, UC: 145	Primary non-response: 29.6 [13.4], secondary non-response: 24.9 [13.8], durable response: 26.9 [13.4]	Caucasian > 84% (NR)	Yes	IFX, ADL, and CLZ for CD; IFX, ADL, and GOL for UC	A: persistence of response	HR: 1.54 (1.27–1.87), *p* = 1.10 × 10^−5^	
***LY96***									
rs11465996	Bank et al. (2014) [18]	IBD: 738, CD: 482, UC: 256	NR [04–81]	NR (Denmark)	NR	Anti-TNF	C: response in combined IBD, CD, and UC	OR: 1.48 (1.00–2.19), *p* = 0.05 (IBD)	
	Salvador-Martín et al. (2019) A [32]	IBD: 210, CD: 147, UC: 63	NR (<18 years)	NR (Spain)	NR	IFX, ADL	C: greater persistence in UC	HR: 5.5 (1.1–28.1), *p* = 0.042	
	Salvador-Martín et al. (2019) A [32]	IBD: 210, CD: 147, UC: 63	NR (<18 years)	NR (Spain)	NR	IFX, ADL	C: higher TL	*p* = 0.002	
	Salvador-Martín et al. (2020) [19]	IBD: 209, CD: 147, UC: 62	10.6 [0.7–17.3]	NR (Spain)	Yes	IFX, ADL	C: persistence of response in UC	HR: 6.052 (1.216–30.125), *p* = 0.028.	
	Salvador-Martín et al. (2021) B [82]	IBD: 154, CD: 106, UC: 48	10.85 [1.5–17.3]	NR (Spain)	Yes	IFX, ADL	CC: lower IFX TL	OR: 0.241 (0.098–0.592), *p* = 0.006	
***MAP3K14***									
rs7222094	Bank et al. (2014) [18]	IBD: 738, CD: 482, UC: 256	NR [04–81]	NR (Denmark)	NR	Anti-TNF	TC: response in combined IBD	OR: 1.92 (1.00–3.68), *p* = 0.05	10
	Jezernik et al. (2023) [25]	CD: 102	27.4 [NR]	NR (Slovenia)	NR	ADL	CC: non-response	*p* = 0.0359	3
***MIF***									
rs755622	Jezernik et al. (2023) [25]	CD: 102	27.4 [NR]	NR (Slovenia)	NR	ADL	GG: response	*p* = 0.004	3
	Repnik et al. (2019) B [74]	CD: 102	NR	NR (Slovenia)	NR	ADL	Response	*p* = 0.004	12
***MMD***									
rs1465352	Gorenjak et al. (2021) [55]	CD: 84	Responders: 27.2 [12.3], non-responders: 29.2 [12.0]	Caucasian (Slovenia)	NR	ADL	C: non-response	OR: 0.25 (0.11–0.60), *p* < 0.01	
rs4422035	Gorenjak et al. (2021) [55]	CD: 84	Responders: 27.2 [12.3], non-responders: 29.2 [12.0]	Caucasian (Slovenia)	NR	ADL	C: non-response	OR: 0.22 (0.09–0.55), *p* < 0.01	
rs9892429	Gorenjak et al. (2021) [55]	CD: 84	Responders: 27.2 [12.3], non-responders: 29.2 [12.0]	Caucasian (Slovenia)	NR	ADL	T: non-response	OR: 0.25 (0.11–0.60), *p* < 0.01	
rs9893820	Gorenjak et al. (2021) [55]	CD: 84	Responders: 27.2 [12.3], non-responders: 29.2 [12.0]	Caucasian (Slovenia)	NR	ADL	A: non-response	OR: 0.25 (0.11–0.60), *p* < 0.01	
rs11656766	Gorenjak et al. (2021) [55]	CD: 84	Responders: 27.2 [12.3], non-responders: 29.2 [12.0]	Caucasian (Slovenia)	NR	ADL	C: non-response	OR: 0.25 (0.11–0.60), *p* < 0.01	
***MMP25***									
rs117642371	Jung et al. (2019) [65]	CD: 104, UC: 31	Primary responders: 25.85 [10.88], non-responders: 24.52 [10.80]	Asian (Korea)	Yes	IFX	C: non-response	OR: 30.68 (3.49–270), *p* = 2.79 × 10^−6^	
***MSGN1***									
rs34069439	Jung et al. (2019) [65]	CD: 104, UC: 31	Primary responders: 25.85 [10.88], non-responders: 24.52 [10.80]	Asian (Korea)	Yes	IFX	T: non-response	OR: 18.83 (3.66–96.94), *p* = 1.73 × 10^−6^	
***MST1***									
rs3197999	Barber et al. (2016) [31]	CD: 427	25.7 [NR]	NR (USA)	Yes	IFX, ADL, and CLZ	A: non-response	OR: 0.51, *p* = 0.02	
***NF-kB1***									
rs7674004	Zhang et al. (2021) [24]	CD: 206	Responders: 23.0 [18.0–27.6], non-responders: 23.5 [16.8–33.5]	NR (China)	Yes	IFX	GG + AA: non-response	OR: 0.47 (0.23–0.97), *p* = 0.039	6
***NFKBIA***									
rs696	Bank et al. (2019) [20]	IBD: 1045, CD: 587, UC: 458	NR [3–84]	Caucasian (Denmark)	NR	IFX, ADL, GOL	A: response in UC and IBD	OR: 1.45 (1.06–2.00), *p* = 0.02 (UC) OR: 1.25 (1.01–1.54), *p* = 0.04 (IBD)	13
***NFIL3***									
rs4743820	Burke et al. (2018) [33]	UC: 231	29.5 [NR]	Caucasian (NR)	Yes	Anti-TNF	C: non-response	OR: 1.81, *p* = 0.044	2
***NLRP3***									
rs10754558	Bank et al. (2018) [73]	CD: 482, UC: 256	NR [04–77]	Caucasian (Denmark)	NR	Anti-TNF	G: response in combined IBD patients	OR: 1.60 (1.02–2.52), *p* = 0.04	11
rs4612666	Bank et al. (2019) [20]	IBD: 1045, CD: 587, UC: 458	NR [3–84]	Caucasian (Denmark)	NR	IFX, ADL, GOL	T: non-response in UC and combined IBD	OR: 0.63 (0.44–0.91), *p* = 0.01 (UC) OR: 0.73 (0.57–0.95), *p* = 0.02 (IBD)	13
	Jezernik et al. (2023) [25]	CD: 102	27.4 [NR]	NR (Slovenia)	NR	ADL	CC: non-response	*p* = 0.03	2
***NR12***									
rs3814057	Koder et al. (2015) [17]	CD: 97	41.1 [11.9]	NR (Slovenia)	Yes	ADL	C: response	OR: 9.07 (1.10–74.49), *p* = 0.00253	4
***NOD2***									
rs2066844	Juanola et al. (2015) [83]	CD: 57	Non-intensified: 43.4 [17.89], intensified: 40.68 [15.54]	Caucasian (Spain)	NR	Anti-TNF	T: loss of response	*p* < 0.01	
rs2066845	Juanola et al. (2015) [83]	CD: 57	Non-intensified: 43.4 [17.89], intensified: 40.68 [15.54]	Caucasian (Spain)	NR	Anti-TNF	C: loss of response	*p* < 0.01	
rs2066847	Juanola et al. (2015) [83]	CD: 57	Non-intensified: 43.4 [17.89], intensified: 40.68 [15.54]	Caucasian (Spain)	NR	Anti-TNF	C: loss of response	*p* < 0.01	
rs2066844, rs2066845, rs2066847	Schäffler et al. (2018) [37]	CD: 29	Variant: 30.7 [13.0], WT: 31.1 [13.5]	NR (Germany)	NR	IFX, ADL	NOD2 MUT: lower TL	*p* = 0.016; IFX *p* = 0.038; ADL *p* = 0.033	
rs5743289	Burke et al. (2018) [33]	UC: 231	29.5 [NR]	Caucasian (NR)	Yes	Anti-TNF	T: better persistence of response in UC	OR: 1.79, *p* = 0.033	2
*p*.Arg702Trp, *p*.Gly908Arg, *p*.Leu1007fsX1008	Niess et al. (2012) [84]	CD: 185	NOD2−/−: 30.1 [14–59], NOD2+/−: 26.4 [15–48], NOD2+/+: 18[NR]	NR (Germany)	NR	IFX, ADL	WT: response	*p* = 0.07	
***NRP1; PARD3***									
rs6481864	Yoon et al. (2017) [48]	CD: 314, UC: 145	Primary non-response: 29.6 [13.4], secondary non-response: 24.9 [13.8], durable response: 26.9 [13.4]	Caucasian > 84% (NR)	Yes	IFX, ADL, and CLZ for CD; IFX, ADL, and GOL for UC	G: persistence of response	HR: 2.31 (1.54–3.48), *p* = 5.19 × 10^−5^	
***OSMR***									
rs357291	Zhang et al. (2021) [24]	CD: 206	Responders: 23.0 [18.0–27.6], non-responders: 23.5 [16.8–33.5]	NR (China)	Yes	IFX	A: primary non-response	OR: 0.33 (0.15–0.73), *p* = 0.005	6
***PDE3A***									
rs3794271	Jezernik et al. (2023) [25]	CD: 102	27.4 [NR]	NR (Slovenia)	NR	ADL	TT: non-response	*p* = 0.0440	3
***PDGFB, RPL3***									
imm_22_37989719	Zhu et al. (2017) [52]	CD: 485, UC: 172	NR	NR	NR	Anti-TNF	Non-response	OR: 3.7, *p* = 0.011	
***PHACTR3***									
rs6100556	Zapata-Cobo (2023) [35]	CD: 240, UC: 93, IC: 7	11.2 [0.7–17.3]	NR (Spain)	Yes	IFX, ADL	TT: loss of response	OR: 1.853 (1.047–3.280) *p* = 0.031 (IBD) OR: 2.997 (1.371–6.553) *p* = 0.04 (UC)	
***PHTF1***									
rs6679677	Burke et al. (2018) [33]	UC: 231	29.5 [NR]	Caucasian (NR)	Yes	Anti-TNF	A: non-response	OR: 2.26, *p* = 0.041	2
***PLA2G4A***									
rs10798069	Zhu et al. (2017) [52]	CD: 485, UC: 172	NR	NR	NR	Anti-TNF	ADA development	*p* = 0.019	
***PLCL1***									
rs1440088	Barber et al. (2016) [31]	CD: 427	25.7 [NR]	NR (USA)	Yes	IFX, ADL, and CLZ	G: persistence of response in CD	OR: 1.74, *p* = 0.04	
1 kg_2_199314605	Zhu et al. (2017) [52]	CD: 485, UC: 172	NR	NR	NR	Anti-TNF	Non-response	OR: 3.4, *p* = 0.044	
***PLIN2***									
rs2228416	Wang et al. (2019) [78]	IBD: 474, CD: 359, UC: 99, IC: 16	Responders: 29.8 [13.4], non-responders: 26.4 [12.1]	NR (European countries)	Yes	IFX, ADL	T: non-response	ORa: 5.25 (2.33–11.8), *p* = 5.24 × 10^−6^	14
***PPBP, CXCL5***									
rs2457996	Zhu et al. (2017) [52]	CD: 485, UC: 172	NR	NR	NR	Anti-TNF	Non-response	OR: 3.4, *p* = 0.035	
***PRDM1; ATG5***									
rs62421049	Yoon et al. (2017) [48]	CD: 314, UC: 145	Primary non-response: 29.6 [13.4], secondary non-response: 24.9 [13.8], durable response: 26.9 [13.4]	Caucasian > 84% (NR)	Yes	IFX, ADL, and CLZ for CD; IFX, ADL, and GOL for UC	T: persistence of response	HR: 1.83 (1.39–2.40), *p* = 1.68 × 10^−5^	
***PREP, PRDM1***									
imm_6_106541962	Zhu et al. (2017) [52]	CD: 485, UC: 172	NR	NR	NR	Anti-TNF	Non-response	OR: 2.6, *p* = 0.047	
***PTGER4***									
rs10512734	Koder et al. (2015) [17]	CD: 97	41.1 [11.9]	NR (Slovenia)	Yes	ADL	GG: positive response	OR: 0.635 (0.541–0.746), *p* = 0.0276	4
***PTPN2***									
rs7234029	Hoffmann et al. (2021) [85]	CD: 110	27.6 [11.9]	NR (Germany)	NR	UST	G: non-response	*p* = 0.005	
rs1893217	Spalinger et al. (2016) [86]	IBD: 1.843, CD: 1073, UC: 734, IC: 46	CD: 25.6 [NR], UC: 30.9 [NR]	NR (European countries 80.65%)	NR	IFX, ADL, CLZ	CT: response	*p* = 0.05	
***PTPRC***									
rs10919563	Jezernik et al. (2023) [25]	CD: 102	27.4 [NR]	NR (Slovenia)	NR	ADL	A: non-response	*p* = 0.0327	2
***RAB38***									
rs9144	Jung et al. (2019) [65]	CD: 104, UC: 31	Primary responders: 25.85 [10.88], non-responders: 24.52 [10.80]	Asian (Korea)	Yes	IFX	A: loss of response	OR: 3.81 (2.10–6.89), *p* = 4.60 × 10^−6^	
***RalGDS/AF-6***									
rs2682714	Barber et al. (2016) [31]	CD: 427	25.7 [NR]	NR (USA)	Yes	IFX, ADL, and CLZ	C: persistence of respons	OR: 2.44, *p* < 0.01	
***RBP-J***									
rs4692386	Burke et al. (2018) [33]	UC: 231	29.5 [NR]	Caucasian (NR)	Yes	Anti-TNF	T: better persistence of response in UC	OR: 0.57, *p* = 0.026	2
***RHCG***									
rs2289352	Yoon et al. (2017) [48]	CD: 314, UC: 145	Primary non-response: 29.6 [13.4], secondary non-response: 24.9 [13.8], durable response: 26.9 [13.4]	Caucasian > 84% (NR)	Yes	IFX, ADL, and CLZ for CD; IFX, ADL, and GOL for UC	A: primary non-response	OR: 2.45 (1.60–3.74), *p* = 3.46 × 10^−5^	
***RIPK1***									
rs9378763	Zhang et al. (2021) [24]	CD: 206	Responders: 23.0 [18.0–27.6], non-responders: 23.5 [16.8–33.5]	NR (China)	Yes	IFX	A: primary non-response	OR: 2.11 (1.00–4.48), *p* = 0.047	6
***RIT1***									
rs70523	Burke et al. (2018) [33]	UC: 231	29.5 [NR]	Caucasian (NR)	Yes	Anti-TNF	A: persistence of response in UC	OR: 0.64, *p* = 0.021	2
***RORB* and *TRPM6***									
rs523781	Wang et al. (2019) [78]	IBD: 474, CD: 359, UC: 99, IC: 16	Responders: 29.8 [13.4], non-responders: 26.4 [12.1]	NR (European countries)	Yes	IFX, ADL	G: non-response	ORa: 3.97 (1.95–8.07), *p* = 5.28 × 10^−5^	14
***RORC***									
rs4845604	Barber et al. (2016) [31]	CD: 427	25.7 [NR]	NR (USA)	Yes	IFX, ADL, and CLZ	A: non-response	OR: 2.46, *p* < 0.01	
***RSP03***									
rs2503322	Barber et al. (2016) [31]	CD: 427	25.7 [NR]	NR (USA)	Yes	IFX, ADL, and CLZ	A: non-response	OR: 0.59, *p* = 0.04	
***RPS6KC1***									
rs144256942	Wang et al. (2019) [78]	IBD: 474, CD: 359, UC: 99, IC: 16	Responders: 29.8 [13.4], non-responders: 26.4 [12.1]	NR (European countries)	Yes	IFX, ADL	G: non-response	ORa: 9.88 (2.88–33.9), *p* = 1.14 × 10^−5^	14
***S100A8*-*S100A9*-*S100A12***									
rs11205276, rs3014866, rs724781, and rs3006488	Medrano et al. (2015) [64]	CD: 350	Responders: 39.6 [0.7], non-responders: 41.9 [1.5]	NR (Spain)	Yes	IFX	GCCA haplotype: non-response	OR: 1.91 (1.05–3.48), *p* = 0.025	
***SFMBT1***									
rs9847710	Barber et al. (2016) [31]	CD: 427	25.7 [NR]	NR (USA)	Yes	IFX, ADL, and CLZ	C: non-response	OR: 0.51, *p* = 0.01	
***SH2B1***									
rs7201929	Barber et al. (2016) [31]	CD: 427	25.7 [NR]	NR (USA)	Yes	IFX, ADL, and CLZ	C: persistence of response	OR: 0.45, *p* = 0.01	
***SLCO1C1***									
rs3794271	Laserna et al. (2023) [47]	CD: 131	36.4 [27.7–46.0]	Caucasian (Spain)	NR	IFX	CC: persistence of response	ORa: 0.2 (0.1–0.7), *p* = 0.008	1
***SLIT1***									
rs7093856	Yoon et al. (2017) [48]	CD: 314, UC: 145	Primary non-response: 29.6 [13.4], secondary non-response: 24.9 [13.8], durable response: 26.9 [13.4]	Caucasian > 84% (NR)	Yes	IFX, ADL, and CLZ for CD; IFX, ADL, and GOL for UC	A: persistence of response	HR: 2.14 (1.48–3.09), *p* = 5.30 × 10^−5^	
***SMAD3***									
rs17293632	Yoon et al. (2017) [48]	CD: 314, UC: 145	Primary non-response: 29.6 [13.4], secondary non-response: 24.9 [13.8], durable response: 26.9 [13.4]	Caucasian > 84% (NR)	Yes	IFX, ADL, and CLZ for CD; IFX, ADL, and GOL for UC	A: persistence of response	HR: 0.69 (0.55–0.86), *p* = 0.0008	
***SMURF1***									
rs9297145	Barber et al. (2016) [31]	CD: 427	25.7 [NR]	NR (USA)	Yes	IFX, ADL, and CLZ	C: persistence of response	OR: 1.57, *p* = 0.05	
***SOCS1, LITAF, RMI2***									
rs529866	Burke et al. (2018) [33]	UC: 231	29.5 [NR]	Caucasian (NR)	Yes	Anti-TNF	T: better persistence of response in UC	OR: 2.18, *p* = 0.039	2
	Zhu et al. (2017) [52]	CD: 485, UC: 172	NR	NR	NR	Anti-TNF	Non-response	OR: 3.6, *p* = 0.009	
***SP140***									
rs6716753	Burke et al. (2018) [33]	UC: 231	29.5 [NR]	Caucasian (NR)	Yes	Anti-TNF	T: better persistence of response in UC	OR: 0.61, *p* = 0.021	2
***STAT3***									
rs744166	Nuij et al. (2017) [49]	CD: 411, UC: 148, ID: 11	27 [5–79]	NR (Netherlands)	NR	IFX, ADL	T: more risk of adverse events with IFX	OR: 0.30 (0.11–0.83), *p* = 0.021	
***ST7, WNT7***									
rs38904	Zhu et al. (2017) [52]	CD: 485, UC: 172	NR	NR	NR	Anti-TNF	ADA development	*p* = 0.040	
***SYNGAP1***									
rs10807124	Yoon et al. (2017) [48]	CD: 314, UC: 145	Primary non-response: 29.6 [13.4], secondary non-response: 24.9 [13.8], durable response: 26.9 [13.4]	Caucasian > 84% (NR)	Yes	IFX, ADL, and CLZ for CD; IFX, ADL, and GOL for UC	A: primary non-response	OR: 2.13 (1.47–3.07), *p* = 5.62 × 10^−5^	
***TAGAP***									
rs212388	Barber et al. (2016) [31]	CD: 427	25.7 [NR]	NR (USA)	Yes	IFX, ADL, and CLZ	C: persistence of response	OR: 0.62, *p* = 0.01	
***TBC1D5***									
rs17200795	Barber et al. (2016) [31]	CD: 427	25.7 [NR]	NR (USA)	Yes	IFX, ADL, and CLZ	G: non-response	OR: 3.19, *p* < 0.01	
***TBX21***									
rs17250932	Bank et al. (2018) [73]	CD: 482, UC: 256	NR [04–77]	Caucasian (Denmark)	NR	Anti-TNF	CC: non-response in UC	OR: 0.06 (0.01–0.80), *p* = 0.03	11
***TLR2***									
rs1816702	Bank et al. (2014) [18]	IBD: 738, CD: 482, UC: 256	NR [04–81]	NR (Denmark)	NR	Anti-TNF	T: response in CD	OR: 2.02 (1.04–3.95), *p* = 0.01	
	Salvador-Martín et al. (2019) A [32]	IBD: 210, CD: 147, UC: 63	NR (<18 years)	NR (Spain)	NR	IFX, ADL	TLR2: serum levels of anti-TNF TL in children with CD	*p* = 0.009	
	Salvador-Martín et al. (2019) B [34]	CD: 132	27.4 [10.8–76.7]	NR (Spain)	Yes	IFX	CC: persistence of response	HR: 0.13 (0.02–1.00), *p* = 0.049	
	Salvador-Martín et al. (2021) B [82]	IBD: 154, CD: 106, UC: 48	10.85 [1.5–17.3]	NR (Spain)	Yes	IFX, ADL	T: higher TL	OR: 0.131 (0.023–0.753), *p* = 0.023	
rs4696480	Bank et al. (2014) [18]	IBD: 738, CD: 482, UC: 256	NR [04–81]	NR (Denmark)	NR	Anti-TNF	TT: non-response in UC	OR: 0.47 (0.23–0.95), *p* = 0.04	10
	Jezernik et al. (2023) [25]	CD: 102	27.4 [NR]	NR (Slovenia)	NR	ADL	AA: non-response	*p* = 0.00148	3
rs11938228	Bank et al. (2014) [18]	IBD: 738, CD: 482, UC: 256	NR [04–81]	NR (Denmark)	NR	Anti-TNF	A: non-response in combined IBD and UC	OR: 0.63 (0.41–0.98), *p* = 0.04 (IBD)	
	Bank et al. (2019) [20]	IBD: 1045, CD: 587, UC: 458	NR [3–84]	NR (Denmark)	NR	IFX, ADL, GOL	AA: non-response in UC	OR: 0.55 (0.33–0.92), *p* = 0.02	13
	Jezernik et al. (2023) [25]	CD: 102	27.4 [NR]	NR (Slovenia)	NR	ADL	CC: non-response	*p* = 0.00982	3
Haplotype 22 rs4696480TT rs1816702CC rs11938228AA rs3804099TT	Bank et al. (2014) [18]	IBD: 738, CD: 482, UC: 256	NR [04–81]	NR (Denmark)	NR	Anti-TNF	Haplotype: non-response	OR: 0.41 (0.19–0.86), *p* = 0.02	10
Haplotype12 rs4696480TA rs1816702CC rs11938228CA rs3804099CT	Bank et al. (2014) [18]	IBD: 738, CD: 482, UC: 256	NR [04–81]	NR (Denmark)	NR	Anti-TNF	Haplotype: non-response	OR: 0.48 (0.24–0.95), *p* = 0.04	10
rs3804099	Bank et al. (2014) [18]	IBD: 738, CD: 482, UC: 256	NR [04–81]	NR (Denmark)	NR	Anti-TNF	C: response in combined IBD, CD, and UC	OR: 1.80 (1.15–2.81), *p* = 0.01 (IBD)	10
	Hu et al. (2021) [41]	CD: 62	11.00 [8.00–12.41]	NR (China)	NR	IFX	C: higher TL		
	Jezernik et al. (2023) [25]	CD: 102	27.4 [NR]	NR (Slovenia)	NR	ADL	CC: non-response	*p* = 0.0389	3
	Salvador-Martín et al. (2019) A [32]	IBD: 210, CD: 147, UC: 63	NR (<18 years)	NR (Spain)	NR	IFX, ADL	TLR2: serum levels of anti-TNF TL in children with CD		
	Salvador-Martín et al. (2019) B [34]	CD: 132	27.4 [10.8–76.7]	NR (Spain)	Yes	IFX	TT: persistence of response	HR: 0.39 [0.18–0.88], *p* = 0.023	
	Stavrou et al. (2022) [87]	CD: 109	45 [11.27]	NR (Greece)	Yes	IFX, ADL	T: non-response	OR: 0.39(0.198–0.747), *p* = 0.003; IFX: 0.46 (0.46–0.94), *p* = 0.032, ADL: 0.166 (0.162–0.969), *p* = 0.026	
***TLR4***									
rs5030728	Bank et al. (2014) [18]	IBD: 738, CD: 482, UC: 256	NR [04–81]	NR (Denmark)	NR	Anti-TNF	A: response in combined IBD, CD, and UC	OR: 1.45 (1.06–2.00), *p* = 0.02 (IBD)	
	Bank et al. (2019) [20]	IBD: 1045, CD: 587, UC: 458	NR [3–84]	NR (Denmark)	NR	IFX, ADL, GOL	AA: response in UC and IBD	OR: 2.23 (1.24–4.01), *p* = 0.01 (UC) 1.46 (1.01–2.11), *p* = 0.04 (IBD)	13
	Salvador-Martín et al. (2019) A [32]	IBD: 210, CD: 147, UC: 63	NR (<18 years)	NR (Spain)	NR	IFX, ADL	Higher TL	*p* = 0.026	
	Salvador-Martín et al. (2021) B [82]	IBD: 154, CD: 106, UC: 48	10.85 [1.5–17.3]	NR (Spain)	Yes	IFX, ADL	GG: lower IFX TL	OR: 3.434 (1.354–8.714), *p* = 0.02	
rs1554973	Bank et al. (2014) [18]	IBD: 738, CD: 482, UC: 256	NR [04–81]	NR (Denmark)	NR	Anti-TNF	C: non-response in combined IBD	OR: 0.72 (0.52–0.99), *p* = 0.04 (IBD)	10
	Bank et al. (2019) [20]	IBD: 1045, CD: 587, UC: 458	NR [3–84]	NR (Denmark)	NR	IFX, ADL, GOL	CC: non-response in UC and IBD	OR: 0.49 (0.27–0.90), *p* = 0.02 (UC) OR: 0.80 (0.65–0.98), *p* = 0.03 (IBD)	13
	Jezernik et al. (2023) [25]	CD: 102	27.4 [NR]	NR (Slovenia)	NR	ADL	C: non-response	*p* = 0.0136	2
***TLR5***									
rs5744174	Bank et al. (2018) [73]	CD: 482, UC: 256	NR [04–77]	Caucasian (Denmark)	NR	Anti-TNF	TC: non-response in CD	OR: 0.36 (0.16–0.81), *p* = 0.01	11
***TLR9***									
rs352139	Bank et al. (2014) [18]	IBD: 738, CD: 482, UC: 256	NR [04–81]	NR (Denmark)	NR	Anti-TNF	AA: non-response in combined IBD and CD	OR: 0.48 (0.24–0.96), *p* = 0.04 (IBD)	10
	Jezernik et al. (2023) [25]	CD: 102	27.4 [NR]	NR (Slovenia)	NR	ADL	AA: non-response	*p* = 0.0198	3
rs187084	Bank et al. (2014) [18]	IBD: 738, CD: 482, UC: 256	NR [04–81]	NR (Denmark)	NR	Anti-TNF	TC: Response in combined IBD	OR: 1.99 (1.04–3.82), *p* = 0.04 (IBD)	10
***TNF***									
rs1799724	Matsuoka et al. (2018) [88]	CD: 121	37.5 [9.5]	NR (Japan)	Yes	IFX	CC: response	ORa: 0.33 (0.12–0.95), *p* = 0.04	
rs1800629	Cheli et al. (2023) [46]	CD: 56, UC: 23	13 [2.5]	NR (Italy)	Yes	IFX, ADL	GG: response to IFX	OR: 12.6 (1.28–124.5), *p* = 0.03 (IFX)	
	Lopez-Hernandez et al. (2014) [89]	CD: 54, UC: 28	Below 18 years: CD (7, 13%), UC(1, 4%); 18–40 years: CD (28, 52%), UC (13, 46%); above 40 years: CD (19, 35%), UC: (14, 50%)	NR (Spain)	NR	IFX, ADL	A: non-response	*p* < 0.05	
	Netz et al. (2017) [29]	CD: 121	41.6 [95% CI: 39.2–44.0]	Caucasian 93% (USA)	Yes	Anti-TNF	A: non-response	OR: 2.88 (1.01–8.22), *p* = 0.049	
	Jezernik et al. (2023) [25]	CD: 102	27.4 [NR]	NR (Slovenia)	NR	ADL	GG: non-response	*p* = 0.00615	3
rs361525	Bank et al. (2014) [18]	IBD: 738, CD: 482, UC: 256	NR [04–81]	NR (Denmark)	NR	Anti-TNF	GA: non-response in combined IBD	OR: 0.43 (0.19–0.97), *p* = 0.04 (IBD)	
	Jezernik et al. (2023) [25]	CD: 102	27.4 [NR]	NR (Slovenia)	NR	ADL	A: non-response	*p* = 0.00279	3
***TNFAIP3***									
rs6927172	Bank et al. (2014) [18]	IBD: 738, CD: 482, UC: 256	NR [04–81]	NR (Denmark)	NR	Anti-TNF	G: non-response in combined IBD and UC	OR: 0.62 (0.42–0.92), *p* = 0.02 (IBD)	10
	Jezernik et al. (2023) [25]	CD: 102	27.4 [NR]	NR (Slovenia)	NR	ADL	CC: non-response	*p* = 0.0387	2
	Salvador-Martín et al. (2019) A [32]	IBD: 210, CD: 147, UC: 63	NR (<18 years)	NR (Spain)	NR	IFX, ADL	Higher TL	*p* = 0.045	
***TNFAIP6***									
rs11677200, rs2342910, rs3755480, rs10432475	Medrano et al. (2015) [64]	CD: 350	Responders: 39.6 [0.7], non-responders: 41.9 [1.5]	NR (Spain)	Yes	IFX	CAGA haplotype: non-response	OR: 0.71 (0.46–1.09), *p* = 0.10	
***TNFRSF1A***									
rs4149570	Bank et al. (2014) [18]	IBD: 738, CD: 482, UC: 256	NR [04–81]	NR (Denmark)	NR	Anti-TNF	TT: response in combined IBD	OR: 2.07 (1.03–4.15), *p* = 0.04 (IBD)	10
	Bank et al. (2019) [20]	IBD: 1045, CD: 587, UC: 458	NR [3–84]	NR (Denmark)	NR	IFX, ADL, GOL	T: response in CD	OR: 1.92 (1.02–3.60), *p* = 0.04	13
rs767455	Jezernik et al. (2023) [25]	CD: 102	27.4 [NR]	NR (Slovenia)	NR	ADL	CC: non-response	*p* = 0.0394	3
	Matsukura et al. (2008) [90]	CD: 80	Responders: 29.9 [7.5], non-responders: 33.7 [9.7]	Asian (Japan)	Yes	IFX	AG: reduced effect	OR: 0.31 (0.11–0.82), *p* = 0.04	
	Pierik et al. (2004) [91]	CD: 344, UC: 152	42.4 [18–76]	NR (Belgium)	Yes	IFX	G: non-response	OR: 0.47 (0.234, 0.946), *p* = 0.0343	
rs1800693	Jezernik et al. (2023) [25]	CD: 102	27.4 [NR]	NR (Slovenia)	NR	ADL	CC: non-response	*p* = 0.0290	
***TNFRSF1B***									
rs3397	Hu et al. (2021) [41]	CD: 62	11.00 [8.00–12.41]	NR (China)	NR	IFX	CC: ADA development	OR: 0.23 (0.06–0.94), *p* = 0.041	
	Matsukura et al. (2008) [90]	CD: 80	Responders: 29.9 [7.5], non-responders: 33.7 [9.7]	Asian (Japan)	Yes	IFX	Haplotype AT: response	*p* = 0.01	
	Medrano et al. (2014) [21]	CD: 297	Non-responders: 43.1 [1.6], responders: 39.9 [0.8]	Caucasian (Spain)	No	IFX	CC: response	OR: 3.19 (0.95–16.78), *p* = 0.05	
	Salvador-Martín et al. (2021) B [82]	IBD: 154, CD: 106, UC: 48	10.85 [1.5–17.3]	NR (Spain)	Yes	IFX, ADL	T: Lower ADL TL	OR: 0.045 (0.003–0.75), *p* = 0.031	
rs1061622	Medrano et al. (2014) [21]	CD: 297	Non-responders: 43.1 [1.6], responders: 39.9 [0.8]	Caucasian (Spain)	Yes	IFX	G: response	*p* = 0.033	
	Jezernik et al. (2023) [25]	CD: 102	27.4 [NR]	NR (Slovenia)	NR	ADL	GG: non-response	*p* = 0.0325	3
	Steenholdt et al. (2012) [22]	CD: 104	Primary non-response: 22 [19–42], loss of response: 24 [20–38], maintained remission: 25 [20–32]	Caucasian (NR)	Yes	IFX	G: response	OR: 4.2 (1.2–18.2), *p* = 0.014	
	Steenholdt et al. (2012) [22]	CD: 104	Primary non-response: 22 [19–42], loss of response: 24 [20–38], maintained remis-sion: 25 [20–32]	Caucasian (NR)	Yes	IFX	G: persistence of response	OR: 5.5 (1.5–25.5), *p* = 0.007	
rs1061624	Matsukura et al. (2008) [90]	CD: 80	Responders: 29.9 [7.5], non-responders: 33.7 [9.7]	Asian (Japan)	Yes	IFX	AT haplotype: response	*p* = 0.01	
	Medrano et al. (2014) [21]	CD: 297	Non-responders: 43.1 [1.6], responders: 39.9 [0.8]	Caucasian (Spain)	No	IFX	A: non-response	OR: 1.63 (1.05–2.51), *p* = 0.02	
	Salvador-Martín et al. (2019) B [34]	CD: 132	27.4 [10.8–76.7]	NR (Spain)	Yes	IFX	A: persistence of response	HR: 0.041 (0.18–0.92), *p* = 0.03	
Haplotype rs1061624, rs3397	Medrano et al. (2014) [21]	CD: 297	Non-responders: 43.1 [1.6], responders: 39.9 [0.8]	Caucasian (Spain)	No	IFX	Haplotype rs1061624/A-rs3397/T: non-response	OR: 1.78 (1.09–2.9), *p* = 0.015	
rs5746053	Walczak et al. (2020) [27]	CD: 107	28 [10.64]	NR (Poland)	No	IFX, ADL	A: response	OR: 0.09 (0.005–1.560), *p* = 0.02	9
rs652625	Steenholdt et al. (2012) [22]	CD: 104	Primary non-response: 22 [19–42], loss of response: 24 [20–38], maintained remission: 25 [20–32]	Caucasian (NR)	Yes	IFX	A: less risk of adverse events with IFX	OR: 0.2, *p* = 0.043	
rs976881	Steenholdt et al. (2012) [22]	CD: 104	Primary non-response: 22 [19–42], loss of response: 24 [20–38], maintained remission: 25 [20–32]	Caucasian (NR)	Yes	IFX	A: loss of response	OR: 3.3 (1.2–9.1), *p* = 0.014	
rs1061628	Smithberger et al. (2014) [92]	IBD: 84	NR	NR (USA)	NR	Anti-TNF	T: non-response	OR: 4.3, *p* = 0.03	
***TNFRSF9***									
rs3766606	Barber et al. (2016) [31]	CD: 427	25.7 [NR]	NR (USA)	Yes	IFX, ADL, and CLZ	T: non-response	OR: 0.32, *p* = 0.02	
***TNFSF4/18***									
rs116724455	Wang et al. (2019) [78]	IBD: 474, CD: 359, UC: 99, IC: 16	Responders: 29.8 [13.4], non-responders: 26.4 [12.1]	NR (European countries)	Yes	IFX, ADL	C: non-response	ORa: 19.9 (4.57–86.7), *p* = 4.79 × 10^−8^	14
***TNFSF15***									
rs4246905	Barber et al. (2016) [31]	CD: 427	25.7 [NR]	NR (USA)	Yes	IFX, ADL, and CLZ	T: non-response	OR: 1.66, *p* = 0.05	
rs6478109	Endo et al. (2020) [93]	CD: 119	<20: 39 patients, ≥20: 80 patients	Asian (Japan)	NR	IFX, ADL	Dominant allele: surgery-free survival significantly lower	OR: 4.67 (1.39–22.1), *p* = 0.025	
***TRAF1***									
rs3761847	Jezernik et al. (2023) [25]	CD: 102	27.4 [NR]	NR (Slovenia)	NR	ADL	AA: non-response	*p* = 0.0268	2
***TRAF3IP2***									
rs3851228	Burke et al. (2018) [33]	UC: 231	29.5 [NR]	Caucasian (NR)	Yes	Anti-TNF	T: non-response in UC	OR: 2.33, *p* = 0.027	2
rs1883136	Urabe S. et al. (2015) [75]	CD: 103	Responders: 35.4 [12.9], non-responders: 37.8 [10.3]	NR (Japan)	YES	IFX	C: response	OR: 10.43 (1.603–77.68), *p* = 0.0149	
***TRAP1***									
rs2158962	Park et al. (2019) [94]	CD: 112	NR	Asian (South Korea)	Yes	IFX	AA homozygous: response	OR: 4.94 (2.65–9.24), *p* = 1.35 × 10^−7^	
***TRIB1***									
rs921720	Barber et al. (2016) [31]	CD: 427	25.7 [NR]	NR (USA)	Yes	IFX, ADL, and CLZ	A: non-response	OR: 0.59, *p* = 0.05	
	Burke et al. (2018) [33]	UC: 231	29.5 [NR]	Caucasian (NR)	Yes	Anti-TNF	Persistence of response in UC	OR: 0.67, *p* = 0.035	2
***TRIM21***									
rs2269330	Zhang et al. (2021) [24]	CD: 206	Responders: 23.0 [18.0–27.6], non-responders: 23.5 [16.8–33.5]	NR (China)	Yes	IFX	G: primary non-response	OR: 0.35 (0.16–0.75), *p* = 0.006	6
***UBAC2, GPR18***									
rs3742130	Burke et al. (2018) [33]	UC: 231	29.5 [NR]	Caucasian (NR)	Yes	Anti-TNF	A: non-response in UC	OR: 1.98, *p* = 0.023	2
***UBE2L3***									
imm_22_20252904	Zhu et al. (2017) [52]	CD: 485, UC: 172	NR	NR	NR	Anti-TNF	ADA development	*p* < 0.001	
***XBP1***									
rs35873774	Nuij et al. (2017) [49]	CD: 411, UC: 148, IC: 11	27 [5–79]	NR (Netherlands)	NR	IFX, ADL	C: non-response to IFX	OR: 3.7 (1.2–10.8), *p* = 0.016	
***ZNF133***									
rs2228273	Jung et al. (2019) [65]	CD: 104, UC: 31	Primary responders: 25.85 [10.88], non-responders: 24.52 [10.80]	Asian (Korea)	Yes	IFX	A: non-response	OR: 11.937 (3.812–37.385), *p* = 2.10 × 10^−5^	
rs34099160	Jung et al. (2019) [65]	CD: 104, UC: 31	Primary responders: 25.85 [10.88], non-responders: 24.52 [10.80]	Asian (Korea)	Yes	IFX	T: non-response	OR: 6.11 (2.659–14.46), *p* = 4.70 × 10^−6^	
***ZNF227***									
rs2168989	Yoon et al. (2017) [48]	CD: 314, UC: 145	Primary non-response: 29.6 [13.4], secondary non-response: 24.9 [13.8], durable response: 26.9 [13.4]	Caucasian > 84% (NR)	Yes	IFX, ADL, and CLZ for CD; IFX, ADL, and GOL for UC	C: persistence of response	HR: 0.65 (0.53–0.80), *p* = 4.98 × 10^−5^	
***ZFP36L1***									
rs194749	Barber et al. (2016) [31]	CD: 427	25.7 [NR]	NR (USA)	Yes	IFX, ADL, and CLZ	C: persistence of response	OR: 1.66, *p* = 0.05	
***ZFP90***									
rs1728785	Barber et al. (2016) [31]	CD: 427	25.7 [NR]	NR (USA)	Yes	IFX, ADL, and CLZ	A: non-response	OR: 0.50, *p* = 0.04	
**Unknown**									
rs2045307	Barber et al. (2016) [31]	CD: 427	25.7 [NR]	NR (USA)	Yes	IFX, ADL, and CLZ	C: non-response	OR: 2.79, *p* < 0.01	
rs10761659	Barber et al. (2016) [31]	CD: 427	25.7 [NR]	NR (USA)	Yes	IFX, ADL, and CLZ	A: non-response	OR: 1.66, *p* = 0.04	
rs8083571	Barber et al. (2016) [31]	CD: 427	25.7 [NR]	NR (USA)	Yes	IFX, ADL, and CLZ	A: non-response	OR: 2.95, *p* < 0.01	
rs2651244	Barber et al. (2016) [31]	CD: 427	25.7 [NR]	NR (USA)	Yes	IFX, ADL, and CLZ	A: persistence of response	OR: 1.51, *p* = 0.04	
rs17119	Barber et al. (2016) [31]	CD: 427	25.7 [NR]	NR (USA)	Yes	IFX, ADL, and CLZ	G: persistence of response	OR: 1.70, *p* = 0.05	
rs904253	Barber et al. (2016) [31]	CD: 427	25.7 [NR]	NR (USA)	Yes	IFX, ADL, and CLZ	A: persistence of response	OR: 0.47, *p* < 0.01	
rs9319943	Burke et al. (2018) [33]	UC: 231	29.5 [NR]	Caucasian (NR)	Yes	Anti-TNF	C: better persistence of response in UC	OR: 1.66, *p* = 0.037	2
rs12051532	Burke et al. (2018) [33]	UC: 231	29.5 [NR]	Caucasian (NR)	Yes	Anti-TNF	C: better persistence of response in UC	OR: 2.35, *p* < 0.01	2
rs1568885	Thomas et al. (2014) [95]	CD: 126	Complete responders: 28.42 [12.85], partial responders: 26.65 [14.21], primary non-responders: 27.32 [13.88]	NR (Greece)	NR	IFX	TT: partial response	OR: 8.14 (1.3549.05) *p* = 0.024	
	Thomas et al. (2014) [95]	CD: 126	Complete responders: 28.42 [12.85], partial responders: 26.65 [14.21], primary non-responders: 27.32 [13.88]	NR (Greece)	NR	IFX	TT: non-response	OR: 21.37 (2.73–167.2), *p* = 0.007	
	Thomas et al. (2014) [95]	CD: 126	Complete responders: 28.42 [12.85], partial responders: 26.65 [14.21], primary non-responders: 27.32 [13.88]	NR (Greece)	NR	IFX	AT: partial response	OR: 2.71 (1.11–6.64), *p* = 0.035	
	Thomas et al. (2014) [95]	CD: 126	Complete responders: 28.42 [12.85], partial responders: 26.65 [14.21], primary non-responders: 27.32 [13.88]	NR (Greece)	NR	IFX	AT: non-response	OR: 4.75 (1.26–17.9), *p* = 0.032	
rs1813443	Thomas et al. (2014) [95]	CD: 126	Complete responders: 28.42 [12.85], partial responders: 26.65 [14.21], primary non-responders: 27.32 [13.88]	NR (Greece)	NR	IFX	CC: partial response	OR: 6.13 (1.74–21.63), *p* = 0.005	
	Thomas et al. (2014) [95]	CD: 126	Complete responders: 28.42 [12.85], partial responders: 26.65 [14.21], primary non-responders: 27.32 [13.88]	NR (Greece)	NR	IFX	CC: non-response	OR: 11.5 (2.5–52.84), *p* = 0.002	

Abbreviations: ADA: anti-drug antibody, ADL: adalimumab, AUC: area under the curve, anti-TNF: anti-tumor necrosis factor, CD: Crohn’s disease, CI: confidence interval, CLZ: certolizumab, CRP: C-reactive protein, GOL: golimumab, HR: hazard ratio, HWE: Hardy–Weinberg equilibrium, IBD: inflammatory bowel disease, IBDQ: Inflammatory Bowel Disease Questionnaire, IC: indeterminate colitis, IFX: infliximab, NR: not reported, OR: odds ratio, ORa: adjusted odds ratio, SD: standard deviation, SNP: single-nucleotide polymorphism, TL: trough level, TL/D: dose-adjusted trough level, USA: United States of America, UST: ustekinumab, VDZ: vedolizumab, VV: valine/valine, WT: wild type, UC: ulcerative colitis. * Observations: 1: This study analyzed 66 SNPs. Only SNPs with significant results were cited. 2: This study analyzed 196,524 SNPs with a multi-SNP-based approach. Only SNPs with significant results were cited. 3: This study analyzed 41 SNPs. Only SNPs with significant results were cited. 4: 33 SNPs were selected for genotyping. Only SNPs with significant results were cited. 5: 28 genetic loci were identified, but only 6 were found to be significantly associated with primary non-response. 6: 125 SNPs within 44 genes were genotyped. Only SNPs with significant results were cited. 7: This study analyzed 540,221 SNPs with a multi-SNP-based approach. Only SNPs with significant results were cited. 8: This study analyzed 77 SNPs with a multi-SNP-based approach. Only SNPs with significant results were cited. 9: This study analyzed 540 SNPs with a multi-SNP-based approach. Only SNPs with significant results were cited. However, after multiple correction, the statistical significance was not rich. 10: This study analyzed 39 SNPs of 26 genes with a multi-SNP-based approach. Only SNPs with significant results were cited. 11: This study analyzed 24 SNPs of 18 genes with a multi-SNP-based approach. Only SNPs with significant results were cited. 12: This study analyzed 45 SNPs. Only SNPs with significant results were cited. 13: This study combines the cohort used by Bank et al. (2014) [18] and Bank et al. (2018) [73] with another new one. 14: GWAIn the column referring to the analyzed study, articles with the same first author and publication year are distinguished by adding a letter (A or B).

This systematic review encompasses a large number of studies on genetic variants that influence the response to biological treatments in patients with IBD. It is a thorough and complex analysis, making it highly relevant due to the growing importance of personalized medicine, which is experiencing exponential development. Despite the comprehensive nature of this analysis, the evidence remains highly variable due to differences in outcomes assessed across studies. Given this diversity, the objective of this review was to synthesize the available evidence in order to identify the genes most likely to impact clinical practice and guide future research efforts in day-to-day patient management.

Polymorphisms in the *ATG16L1* gene have shown differing associations with treatment outcomes. Several studies have found significant associations between certain polymorphisms and better response and persistence in CD patients [17,31,47]. In contrast, other variants have been linked to non-response [23] or loss of response to IFX [35], as well as lower IFX TLs in CD patients [36], suggesting a potential predictive value for treatment failure. These results highlight the complex role of *ATG16L1* polymorphisms in autophagy processes and their potential to influence therapeutic responses in IBD treatment. Further research is needed to validate these associations and establish specific recommendations.

*C1orf106* gene has shown diverging associations with IFX response and pharmacokinetics in CD patients. Some genetic variants might contribute to faster drug clearance and reduced efficacy [24,36]. However, the CC genotype of the rs59457695 variant was linked to higher IFX TLs, indicating a potential beneficial impact on maintaining therapeutic drug levels in these patients [36]. Interestingly, Tang et al. also observed that it is actually the presence of the T allele of this SNP—not the CC genotype—that is associated with clinical response to IFX [36]. This finding implies that, while the CC genotype may contribute to higher drug levels, it is the T allele that correlates with a more effective clinical outcome, highlighting a complex relationship among genotype, drug levels, and treatment response. Overall, these findings emphasize the role of *C1orf106* genetic variants in modulating IFX pharmacokinetics and treatment outcomes in CD patients. Nevertheless, further studies are necessary to clarify their influence across diverse populations and therapeutic contexts.

Bank et al. demonstrated that the A allele in the *CD14* gene’s rs2569190 is associated with non-response in UC patients [18]. In contrast, Jezernik et al. further supported this association but identified the G allele in the same polymorphism as being correlated with non-response to ADL at week 30 in CD patients [25]. These findings highlight the potential role of this polymorphism in predicting non-response to anti-TNF therapy in IBD, making it a relevant variant to evaluate prior to initiating treatment with these medications. However, no significant differences among genotypes were observed in terms of treatment response in studies conducted by Salvador-Martín et al. [19,34,82], except for an association of the A allele of this SNP with lower TLs of anti-TNF drugs in pediatric patients [32], suggesting that the impact of this polymorphism might vary depending on the population or context.

The *FAS* gene polymorphism rs7896789 has been associated with a lack of response to IFX and ADL in CD patients. The findings suggest that the *FAS* gene, which is involved in apoptosis regulation [96], might impact the effectiveness of anti-TNF therapies, suggesting that rs7896789 could be a potential candidate for genetic screening to personalize treatment strategies for CD patients. Correspondingly, the Fas ligand (*FASLG*) gene polymorphism rs763110 also has been linked with worse responses, lower drug TLs, and increased adverse events during IFX treatment [22,28,29,32]. Later studies reported no differences in response between genotypes [19,34,48]. These findings underscore the complexity of genetic influences on treatment response in IBD, suggesting that rs763110 may serve as a valuable marker for personalized treatment strategies, although further investigation is required to confirm this association.

The *FCGR3A* gene encodes the Fc gamma receptor IIIa, which plays a key role in antibody-dependent immune responses. The rs396991 variant appears to be the most studied polymorphism, with multiple studies linking it to ADA development, lower IFX levels, and treatment response [38,39,40,50,51,52,53]. However, results across different contexts are not entirely consistent, particularly in terms of valine/valine (VV) genotype. Romero-Cara et al. confirmed that this genotype was associated with a greater likelihood of ADA formation [40], and Ternant et al. found that the VV genotype was linked to increased IFX elimination and loss of response after discontinuation [59], highlighting the variant’s potential influence on drug metabolism. Conversely, Louis et al. and Moroi et al. reported a positive association between the genotype and response to IFX therapy [56,57,58], and other studies found no association [29,88,97]. This indicates that the effects of this variant may be population-specific or dependent on other genetic or environmental factors, and it suggests that while some polymorphisms show initial promise, they may require larger cohorts or stricter statistical correction to verify their true clinical relevance.

Recently, multiple studies have identified the HLA-DQA1*05 (rs2097432) variant as a predictor of immunogenicity and secondary loss of response to TNF-α compared with non-carriers of this variant [43,45]. These associations suggest that determination of HLA-DQA1*05 variant could serve as a predictive tool to identify patients at higher risk of developing immunogenicity-related complications. Interestingly, studies with proactive TDM protocols reported less pronounced differences in ADA development between carriers and non-carriers of HLA-DQA1*05, indicating that maintaining optimal TLs could mitigate the immunogenicity risk linked to genetic predisposition [46]. This suggests that the increased risk of immunogenicity and loss of response linked to HLA-DQA1*05 might be mitigated by optimizing TLs and proactive TDM.

Variants in genes related to multiple pro-inflammatory interleukins that could affect the response to biological treatments have also been studied. For the rs10499563 variant of the *IL6* gene, the C allele was linked to a greater response to anti-TNF therapy and longer persistence with IFX in CD, as reported by Bank et al. and Salvador-Martín et al. [18,32]. However, other studies [41,48] found no significant response differences by genotype. In a related context, rs1800872 is the genetic variant of greatest interest in the *IL10* gene, given the results shown in different studies. Salvador-Martín et al. identified that the C allele improved treatment persistence [32], and in another study the CC genotype was associated with better responses in CD [19]. Nonetheless, findings from Hu et al. and Salvador-Martín et al. again showed no significant differences in treatment outcomes by genotype, reflecting inconsistencies across studies [41,48].

The rs2275913 variant of the *IL17A* gene has been extensively studied across multiple cohorts of IBD patients, with mixed results in relation to treatment outcomes. Bank et al., in a study with a large sample size of 738 IBD patients, identified that the A allele was associated with non-response to anti-TNF [18], whereas Salvador-Martín et al. reported that the AA genotype was correlated with favorable responses in CD [19]. Jezernik et al., in a Slovenian cohort, showed that the GG genotype was significantly associated with better responses to ADL [25]. However, other studies found no statistically significant differences in treatment response [34,41].

The *IL1B* gene’s rs4848306 variant has shown a significant association with treatment responses in IBD. Bank et al. reported that the A allele was correlated with a positive response to anti-TNF [18], while Jezernik et al. found that the GG genotype was linked to non-response in patients treated with ADL [25]. However, Salvador-Martín et al. did not find significant differences in responses based on this genotype, suggesting that the association may be context-dependent [19,32,34,82]. On the other hand, the rs1143634 polymorphism shows inconsistent findings regarding its association with therapeutic outcomes. Guardiola et al. found that the A allele was linked to lower IFX TLs [76], while Santacana et al. reported that the CC genotype was associated with lower TLs [77]. Similarly, conflicting findings have been reported regarding treatment response in various studies [26,27,30]. These discrepancies may be attributable to population genetics, sample sizes, or treatment protocols, highlighting the need for larger, multicenter studies to clarify the role of rs1143634 in treatment response.

While genetic variants in multiple pro-inflammatory interleukin genes appear to play a role in modulating the response to biological treatments, the variability in findings across studies highlights the complexity of these interactions and underscores the need for further research to clarify their clinical significance.

The *IL1R* gene, which encodes the interleukin-1 receptor, plays a critical role in the inflammatory response by mediating the effects of pro-inflammatory cytokines like IL1. Lykowska-Szuber et al. observed that the rs2041747 G allele was linked to non-response to induction therapy with IFX and ADL in a cohort of 196 CD patients [26]. Similarly, Walczak et al. found that the rs1071676 A allele was associated with non-response in 107 CD patients [27]. In parallel, the *IL1RN* gene, which encodes the IL1 receptor antagonist, plays a regulatory role, and its genetic variants have also been implicated in treatment outcomes. Bank et al. demonstrated that the rs4251961 C allele was associated with non-response to anti-TNF therapy in UC [18], and further studies revealed similar trends across broader IBD populations [20,25].

Polymorphism in the *NOD2* gene alters the receptor that induces alterations on Pleyer’s patch in the small bowel and, consequently, alters the intestinal permeability [98]. Only some studies suggest an association with the variant alleles and their response. Interestingly, rs2066844 (T), rs2066845 (C), and rs2066847 (C), studied by Juanola et al., showed loss of response with variant alleles [83]. Moreover, Schäffler et al. (2018) studied the three previous SNPs in combination, concluding that the variant allele leads to lower TLs [37]. Only the rs5743289 studied by Burke et al. (2018) in UC patients showed a better persistence of anti-TNF drugs [33].

NFκB is an inducible transcription factor that regulates the transcription of various genes involved in inflammation development [99], including *TLR2*, *TLR4*, *TLR9*, and *NFKBIA*, among others. For *TLR2*, the rs1816702 variant allele may induce a better response in CD patients, although the study of Salvador-Martin et al. showed this response in the presence of the dominant allele [34]. The presence of TLR2’s rs11938228 variant allele is related to non-response to the studied anti-TNF, although Jezernik et al. found that non-response was related more to the dominant allele [25]. TLR2’s rs3804099 has been analyzed in several articles, whereas with the heterogeneity of results, no conclusion about the relationship of the SNPs and treatment response can be extracted. For *TLR4*, rs5030728 also shows a diversity of contradictory results. On the other hand, the rs1554973 variant allele could suggest an association with a lack of response. For *TLR9*, the rs352139 variant allele led to non-response in two studies [18,25].

Despite the large number of SNPs studied in TNF, no correlation between an allele and the therapeutic response could be extracted. Nonetheless, for rs1800629, Cheli et al. have shown that the GG genotype has a marked probability of response to IFX [46], although Lopez-Hernandez et al., Netz et al., and Jezernik et al. have found non-response to anti-TNF for the variants A, A, and GG, respectively [25,29,89].

Several studies have demonstrated an influence of TNF receptor SNPs and response to anti-TNF. For *TNFRSF1A* rs4149570, only Bank et al. observed responses to anti-TNF in CD or in a combined IBD cohort [18,20]. For rs767455, Jezernik et al. showed non-response with the CC variant [25], whereas Pierik et al. found this association with the G variant [91].

For *TNFRSF1B* rs3397, it is worth noting the possible association of the CC variant with response to the biological drug shown by Medrano et al. [21], whereas Hu et al. found a more likely possibility of ADA development with the same variant, which is contradictory [41]. For rs1061622, Medrano et al. and Steenholdt et al. observed responses to the drug with the G variant [21,22], although Jezernik et al. found the opposite [25]. For rs1061624, Matsukura et al. found responses for patients with the AT haplotype [90], which is in the same line as the findings of Salvador-Martin et al. with a persistence of response for the A variant [34]. On the other hand, Medrano et al. observed non-response with this same variant [21].

Some SNPs are not related to any gene, like rs1568885 and rs1813443, but are associated with relevant findings. For the former, a variant T allele was significantly associated with partial and non-response to IFX [65], and for the latter, only the C allele of the rs1813443 polymorphism was associated with partial response and non-response, with high probabilities in both cases [65].

Several studies included in this review did not find statistically significant differences in treatment response based on pharmacogenetic profiles [100,101,102,103,104,105,106,107,108,109,110,111,112,113,114,115]. Specific details of these studies are presented in Supplementary Materials (Supplementary Table S1).

## 4. Limitations and Strengths

This systematic review provides a comprehensive overview of the current understanding of genetic variants linked to the response to biological treatments in patients with IBD. To the best of our knowledge, this systematic review includes the largest number of studies and SNPs to date, complementing and expanding the evidence provided by previous authors [116,117,118]. By linking genetic variation to drug response, pharmacogenetic studies help improve our understanding of the mechanisms underlying anti-TNF therapy and how immune and inflammatory pathways are modulated.

The tables that we present summarize the current state of knowledge on pharmacogenetics in IBD and, moreover, highlight potential candidate SNPs as predictive biomarkers of response. These findings may have long-term implications for drug development and clinical guidelines, potentially promoting the broader integration of genetic testing in routine clinical practice for IBD management.

Many of the larger pharmacogenetic studies involve collaborations across multiple centers and countries, increasing the sample size and diversity of the patient populations, thereby improving the robustness of the findings.

Nonetheless, our review presents several limitations, which are mainly due to the high heterogeneity of the published studies. In fact, there are studies with adult and pediatric populations, different ethnic groups, or few SNPs studied several times. Studies often vary significantly in their design, including differences in drug regimens or endpoints measured, and use varying definitions of clinical response or non-response to biological therapy. This inconsistency complicates direct comparisons across studies.

Many pharmacogenetic analyses are limited by small sample sizes, reducing their statistical power and making it difficult to detect significant associations between genetic variants and treatment outcomes. Moreover, some investigations focus solely on the short-term response to anti-TNF therapy, whereas long-term outcomes such as sustained remission, drug discontinuation, or loss of response over time may provide more clinically relevant information.

Moreover, there has been no consideration of environmental factors or concomitant medications that could affect the therapeutic response to the drug, which would allow for extracting reliable conclusions. 

Therefore, further prospective studies are necessary to validate and refine the different responses to biological treatment with the identified SNPs. Additionally, most research has focused on anti-TNF therapies, underscoring the need to expand investigations to other biologics like VDZ and UST.

## 5. Conclusions

The improvements in sequencing techniques have facilitated the study of the genetic variants that may influence the response to biological therapy in IBD patients. Today, the most extensively studied variants are in genes involved in immune regulation, inflammatory pathways, and the metabolism of biologic drugs. However, broader and well-structured studies are still needed to validate these findings and to determine their long-term relevance in patient treatment. Moreover, the complexity of IBD’s pathophysiology and the role of non-genetic factors must be considered. Future research should focus on integrating pharmacogenetics with clinical and environmental factors to develop comprehensive predictive models for treatment success.

The incorporation of pharmacogenetic testing into clinical practice has the potential to revolutionize IBD’s management by providing tailored therapies that optimize efficacy and minimize toxicity, paving the way for more precise and individualized care. While the field of pharmacogenetics holds great promise, more robust evidence is required before routine genetic testing can be integrated into clinical practice.

The conclusions yielded by the present review represent an initial step toward personalized medicine in IBD, which may improve the clinical outcomes with biological therapies. However, significant challenges remain before pharmacogenetics can be fully implemented in routine clinical practice to personalize IBD treatment.

## Supplementary Materials

The supporting information can be downloaded at: https://www.mdpi.com/article/10.3390/ijms26041760/s1.
