# Supplementary material for: Pharmacogenetics in Response to Biological Agents in Inflammatory Bowel Disease: A Systematic Review
Source: Int J Mol Sci. 2025 Feb 19;26(4):1760. doi: 10.3390/ijms26041760 (PMC11855474; doi:10.3390/ijms26041760)
Supplement: Supplementary file 1 [file ijms-26-01760-s001.zip › Suppl.Table S1. Genes and Genetic Variants Not Showing Statistically Significant Associations with Clinical Response to Biological Therapies.pdf]

Supplementary Table S1. Genes and Genetic Variants Not Showing Statistically Significant Associations with Clinical Response to Biological Therapies

SNP	Study	n	Age in years (mean [SD] or median [range])	Ethnia (Country)	HWE	Drug	Clinical outcomes (associated alelle, genotype or haplotype)
**ABCB1**							
rs2032582	Fischer et al. (2007) [100]	CD: 47	33.2 [11.6]	NR (Hungary)	Yes	IFX	No difference between genotypes
rs1045642	Fischer et al. (2007) [100]	CD: 47	33.2 [11.6]	NR (Hungary)	Yes	IFX	No difference between genotypes
**ABCG2**							
rs2231137	Fischer et al. (2007) [100]	CD: 47	33.2 [11.6]	NR (Hungary)	Yes	IFX	No difference between genotypes
rs2231142	Fischer et al. (2007) [100]	CD: 47	33.2 [11.6]	NR (Hungary)	Yes	IFX	No difference between genotypes
**ACTB**							
ACTB	Salvador-Martín et al. (2021) A [61]	IBD:38, CD:30, UC:8	10.5 [0.7–17]	NR (Spain)	NR	IFX, ADL	No difference between genotypes
**ADAM17**							
rs2001658	Dideberg et al. (2006) A [101]	CD: 222	21 [11-31]	Caucasian (NR)	Yes	IFX	No difference between genotypes
rs12469362	Dideberg et al. (2006) A[101]	CD: 222	21 [11-31]	Caucasian (NR)	Yes	IFX	No difference between genotypes
rs883399	Dideberg et al. (2006) A[101]	CD: 222	21 [11-31]	Caucasian (NR)	Yes	IFX	No difference between genotypes
rs1048610	Dideberg et al. (2006)A [101]	CD: 222	21 [11-31]	Caucasian (NR)	Yes	IFX	No difference between genotypes
rs2276338	Dideberg et al. (2006) A[101]	CD: 222	21 [11-31]	Caucasian (NR)	Yes	IFX	No difference between genotypes
rs1056204	Dideberg et al. (2006) A[101]	CD: 222	21 [11-31]	Caucasian (NR)	Yes	IFX	No difference between genotypes
rs10929587	Dideberg et al. (2006) A[101]	CD: 222	21 [11-31]	Caucasian (NR)	Yes	IFX	No difference between genotypes
rs1880439	Dideberg et al. (2006) A[101]	CD: 222	21 [11-31]	Caucasian (NR)	Yes	IFX	No difference between genotypes
rs10495565	Dideberg et al. (2006) A[101]	CD: 222	21 [11-31]	Caucasian (NR)	Yes	IFX	No difference between genotypes
rs4464248	Dideberg et al. (2006) A[101]	CD: 222	21 [11-31]	Caucasian (NR)	Yes	IFX	No difference between genotypes
rs11684747	Dideberg et al. (2006)A [101]	CD: 222	21 [11-31]	Caucasian (NR)	Yes	IFX	No difference between genotypes
rs10929590	Dideberg et al. (2006)A [101]	CD: 222	21 [11-31]	Caucasian (NR)	Yes	IFX	No difference between genotypes
Haplotype rs2001658 rs12469362 rs883399 rs1048610 rs2276338 rs1056204 rs10929587 rs1880439	Dideberg et al. (2006)A [101]	CD: 222	21 [11-31]	Caucasian (NR)	Yes	IFX	No difference between haplotypes

rs10495565<br>rs4464248<br>rs11684747							
rs55790676	Lykowska-Szuber et al. (2023)[26]	CD: 196	30.65 [10.58]	NR (Poland)	Yes	IFX, ADL	No difference between genotypes
**ATG12**							
rs26538	Dezelak et al. (2016)[50]	CD:79	26.6 [12.2]	NR (Slovenia)	NR	ADL	No difference between genotypes
**ATG16L1**							
rs10210302	Netz et al. (2017)[29]	CD: 121	41.6 [95%CI: 39.2-44.0]	Caucasian 93% (USA)	yes	Anti-TNF	No difference between genotypes
rs2241880	Netz et al. (2017) [29]	CD:121	41.6 [95%CI: 39.2-44.0]	Caucasian 93% (USA)	yes	Anti-TNF	No difference between genotypes
rs12616785	Tang et al. (2020) [36]	CD: 189	23 [18–28]	NR (China)	Yes	IFX	No difference between genotypes
rs74660875	Tang et al. (2020) [36]	CD: 189	23 [18–28]	NR (China)	Yes	IFX	No difference between genotypes
rs13032115	Tang et al. (2020) [36]	CD: 189	23 [18–28]	NR (China)	Yes	IFX	No difference between genotypes
rs4663396	Tang et al. (2020) [36]	CD: 189	23 [18–28]	NR (China)	Yes	IFX	No difference between genotypes
rs56805998	Tang et al. (2020) [36]	CD: 189	23 [18–28]	NR (China)	Yes	IFX	No difference between genotypes
rs7587633	Tang et al. (2020) [36]	CD: 189	23 [18–28]	NR (China)	Yes	IFX	No difference between genotypes
**ATG5**							
rs510432	Dezelak et al. (2016)[50]	CD:79	26.6 [12.2]	NR (Slovenia)	NR	ADL	No difference between genotypes
**BIRC5**							
rs1489553229	Rapti et al. (2015) [102]	CD: 97,<br>UC: 88	CD: 23.66 [13.42], UC: 30.01 [14.22]	NR (Greece)	NR	IFX	No difference between genotypes
**C1orf106**							
rs61740234	Tang et al. (2020) [36]	CD: 189	23 [18–28]	NR (China)	Yes	IFX	No difference between genotypes
rs59757713	Tang et al. (2020) [36]	CD: 189	23 [18–28]	NR (China)	Yes	IFX	No difference between genotypes
**CCR6**							
rs2301436	Nuij et al. (2017) [49]	CD: 411,<br>UC:148,<br>IC: 11	27 [5–79]	NR (Netherlands)	NR	IFX, ADL	No difference between genotypes
**CD14**							
rs2569190	Salvador-Martín et al. (2019) B [34]	CD: 132	27.4 [10.8-76.7]	NR (Spain)	Yes	IFX	No difference between genotypes
	Salvador-Martín et al. (2020) [19]	IBD:209,<br>CD: 147,<br>UC:62	10.6 [0.7–17.3]	NR (Spain)	Yes	IFX, ADL	No difference between genotypes
	Salvador-Martín et al. (2021) B [82]	IBD:154,<br>CD:106,<br>UC: 48	10.85 [1.5–17.3]	NR (Spain)	yes	IFX, ADL	No difference between genotypes
	Barreiro-de Acosta et al. (2010)	CD: 24	40.5 [9.58]	NR (Spain)	NR	ADL	No difference between genotypes

	[103]						
**CRP**							
rs1205	Dezelak et al. (2016) [50]	CD:79	26.6 [12.2]	NR (Slovenia)	NR	ADL	No difference between genotypes
Haplotypes rs1205 rs1130864	Dezelak et al. (2016) [50]	CD:79	26.6 [12.2]	NR (Slovenia)	NR	ADL	No difference between genotypes
717G/A	Willot et al. (2006) [104]	CD: 189	34 [16–76]	NR (France)	yes	IFX	No difference between genotypes
1444C/T	Willot et al. (2006) [104]	CD: 189	34 [16–76]	NR (France)	yes	IFX	No difference between genotypes
4A/G	Willot et al. (2006) [104]	CD: 189	34 [16–76]	NR (France)	yes	IFX	No difference between genotypes
**CYP2C9**							
CYP2C9	Wang et al. (2021) [54]	IBD: 51	39 [17.5]	NR (European countries)	Yes	IFX, ADL	No difference between genotypes
**CYP2C19**							
CYP2C19	Wang et al. (2021) [54]	IBD: 51	39 [17.5]	NR (European countries)	Yes	IFX, ADL	No difference between genotypes
**CYP2D6**							
CYP2D6	Wang et al. (2021) [54]	IBD: 51	39 [17.5]	NR (European countries)	Yes	IFX, ADL	No difference between genotypes
**CYP3A4**							
CYP3A4	Wang et al. (2021) [54]	IBD: 51	39 [17.5]	NR (European countries)	Yes	IFX, ADL	No difference between genotypes
**CYP3A5**							
CYP3A5	Wang et al. (2021) [54]	IBD: 51	39 [17.5]	NR (European countries)	Yes	IFX, ADL	No difference between genotypes
**DPYD**							
DPYD	Wang et al. (2021) [54]	IBD: 51	39 [17.5]	NR (European countries)	Yes	IFX, ADL	No difference between genotypes
**FAS**							
rs28362322	Steenholdt et al. (2012) [22]	CD:104	Primary nonresponse: 22 [19–42], Loss of response: 24 [20–38], Maintained remission: 25 [20–32]	Caucasian (NR)	Yes	IFX	No difference between genotypes
rs3218611	Steenholdt et al. (2012) [22]	CD:104	Primary nonresponse: 22 [19–42], Loss of response: 24 [20–38], Maintained remission: 25 [20–32]	Caucasian (NR)	Yes	IFX	No difference between genotypes
**FASLG**							
rs763110	Salvador-Martín et al. (2019) B [34]	CD: 132	27.4 [10.8-76.7]	NR (Spain)	Yes	IFX	No difference between genotypes
	Salvador-Martín et al. (2020) [19]	IBD:209, CD: 147,	10.6 [0.7–17.3]	NR (Spain)	Yes	IFX, ADL	No difference between genotypes

		UC:62					
	Salvador-Martín et al. (2021) B [82]	IBD:154, CD:106, UC: 48	10.85 [1.5–17.3]	NR (Spain)	yes	IFX, ADL	No difference between genotypes
rs75622480	Steenholdt et al. (2012) [22	CD:104	Primary nonresponse: 22 [19–42], Loss of response: 24 [20–38], Maintained remission: 25 [20–32]	Caucasian (NR)	Yes	IFX	No difference between genotypes
rs5030772	Steenholdt et al. (2012) [22]	CD:104	Primary nonresponse: 22 [19–42], Loss of response: 24 [20–38], Maintained remission: 25 [20–32]	Caucasian (NR)	Yes	IFX	No difference between genotypes
**FCGR2A**							
rs1801274	Matsuoka et al. (2018) [88]	CD: 121	37.5 [9.5]	NR (Japan)	yes	IFX	No difference between genotypes
FCGR2A (FcgammaRIIA-131H/R)	Tomita et al. (2010) [60]	CD: 41	30 [18-54]	NR (Japan)	NR	IFX	No difference between genotypes
**FCGR3A**							
rs396991	Netz et al. (2017) [29]	CD:121	41.6 [95%CI: 39.2-44.0]	Caucasian 93% (USA)	Yes	Anti-TNF	No difference between genotypes
	Matsuoka et al. (2018) [88]	CD: 121	37.5 [9.5]	NR (Japan)	Yes	IFX	No difference between genotypes
	Papamichael et al. (2011) [97]	CD: 106	Complete responders: 32 [14.78], Partial responders: 27.33 [13.98], Primary nonresponders: 27.67 [16.58]	NR (Greece)	NR	IFX	No difference between genotypes
	Tomita et al. (2010) [60]	CD: 41	30 [18-54]	NR (Japan)	NR	IFX	No difference between genotypes
rs111504845	Tang et al. (2020) [36]	CD: 189	23 [18–28]	NR (China)	Yes	IFX	No difference between genotypes
rs7539036	Lykowska-Szuber et al. (2023) [26]	CD: 196	30.65 [10.58]	NR (Poland)	Yes	IFX, ADL	No difference between genotypes
rs112142198	Tang et al. (2020) [36]	CD: 189	23 [18–28]	NR (China)	Yes	IFX	No difference between genotypes
rs61801820	Tang et al. (2020) [36]	CD: 189	23 [18–28]	NR (China)	Yes	IFX	No difference between genotypes
rs117758842	Tang et al. (2020) [36]	CD: 189	23 [18–28]	NR (China)	Yes	IFX	No difference between genotypes
rs10917571	Tang et al. (2020) [36]	CD: 189	23 [18–28]	NR (China)	No	IFX	No difference between genotypes
rs114010589	Tang et al. (2020) [36]	CD: 189	23 [18–28]	NR (China)	No	IFX	No difference between genotypes
rs56150752	Tang et al. (2020) [36]	CD: 189	23 [18–28]	NR (China)	No	IFX	No difference between genotypes
rs67295569	Tang et al. (2020) [36]	CD: 189	23 [18–28]	NR (China)	No	IFX	No difference between genotypes
rs35276103	Tang et al. (2020) [36]	CD: 189	23 [18–28]	NR (China)	Yes	IFX	No difference between genotypes
**GBP5**							
GBP5	Salvador-Martín et al. (2021) A [61]	IBD:38, CD:30, UC:8	10.5 [0.7–17]	NR (Spain)	NR	IFX, ADL	No difference between genotypes

**GNLY**							
GNLY	Salvador-Martín et al. (2021) A [61]	IBD:38, CD:30, UC:8	10.5 [0.7–17]	NR (Spain)	NR	IFX, ADL	No difference between genotypes
**HLA-A**							
HLA-A	Wang et al. (2021) [54]	IBD: 51	39 [17.5]	NR (European countries)	Yes	IFX, ADL	No difference between genotypes
**HLA-B**							
HLA-B	Wang et al. (2021) [54]	IBD: 51	39 [17.5]	NR (European countries)	Yes	IFX, ADL	No difference between genotypes
**HLA-DQA1*05**							
rs2097432	Cheli et al. (2023) [46]	CD: 56, UC: 23	13 [2.5]	NR (Italy)	Yes	IFX, ADL	No difference between genotypes
	Hu et al. (2021) [41]	CD: 62	11.00 [8.00-12.41]	NR (china)	NR	IFX	No difference between genotypes
	Laserna et al. (2023) [47]	CD: 131	36.4 [27.7–46.0]	Caucasian (Spain)	NR	IFX	No difference between genotypes
	Spencer et al. (2021) [105]	IBD:186	17 [14-20]	Caucasian 81% (USA)	NR	IFX	No difference between genotypes
**IBD5**							
IGR2060a1 and IGR3081a1	Lu et al. (2012) [106]	CD: 14	Remaining in remission: 30 [14-47], Lost remission: 26 [15-38]	Caucasian (Canada)	NR	IFX	No difference between genotypes
rs2522057	Netz et al. (2017) [29]	CD:121	41.6 [95%CI: 39.2-44.0]	Caucasian 93% (USA)	yes	Anti-TNF	No difference between genotypes
**IGHG1**							
CH1 359g/a	Magdelaine-Beuzelin et al. (2009) [107]	CD:118	NR	Caucasian (European countries)	yes	IFX	No difference between genotypes
**IGHG2**							
IGHG2	Salvador-Martín et al. (2021) A [61]	IBD:38, CD:30, UC:8	10.5 [0.7–17]	NR (Spain)	NR	IFX, ADL	No difference between genotypes
**IL6**							
rs10499563	Hu et al. (2021) [41]	CD: 62	11.00 [8.00-12.41]	NR (china)	NR	IFX	No difference between genotypes
	Salvador-Martín et al. (2021) B [82]	IBD:154, CD:106, UC: 48	10.85 [1.5–17.3]	NR (Spain)	No	IFX, ADL	No difference between genotypes
**IL10**							
rs1800872	Hu et al. (2021) [41]	CD: 62	11.00 [8.00-12.41]	NR (china)	NR	IFX	No difference between genotypes

	Salvador-Martín et al. (2021) B [82]	IBD:154, CD:106, UC: 48	10.85 [1.5–17.3]	NR (Spain)	yes	IFX, ADL	No difference between genotypes
rs3024505	Hu et al. (2021) [41]	CD: 62	11.00 [8.00-12.41]	NR (china)	NR	IFX	No difference between genotypes
	Salvador-Martín et al. (2019) A [32]	IBD:210, CD:147, UC:63	NR (<18 years)	NR (Spain)	NR	IFX, ADL	No difference between genotypes
	Salvador-Martín et al. (2020) [19]	IBD:209, CD: 147, UC:62	10.6 [0.7–17.3]	NR (Spain)	Yes	IFX, ADL	No difference between genotypes
	Salvador-Martín et al. (2021) B [82]	IBD:154, CD:106, UC: 48	10.85 [1.5–17.3]	NR (Spain)	yes	IFX, ADL	No difference between genotypes
**IL17A**							
rs2275913	Hu et al. (2021) [41]	CD: 62	11.00 [8.00-12.41]	NR (china)	NR	IFX	No difference between genotypes
	Salvador-Martín et al. (2019) B [34]	CD: 132	27.4 [10.8-76.7]	NR (Spain)	Yes	IFX	No difference between genotypes
	Salvador-Martín et al. (2021) B [82]	IBD:154, CD:106, UC: 48	10.85 [1.5–17.3]	NR (Spain)	yes	IFX, ADL	No difference between genotypes
	Urabe S. et al. (2015) [75]	CD: 103	Responders: 35.4 [12.9], Nonresponders: 37.8 [10.3]	NR (Japan)	YES	IFX	No difference between genotypes
rs4711998	Urabe S. et al. (2015) [75]	CD: 103	Responders: 35.4 [12.9], Nonresponders: 37.8 [10.3]	NR (Japan)	YES	IFX	No difference between genotypes
rs8193036	Urabe S. et al. (2015) [75]	CD: 103	Responders: 35.4 [12.9], Nonresponders: 37.8 [10.3]	NR (Japan)	YES	IFX	No difference between genotypes
rs3819024	Urabe S. et al. (2015) [75]	CD: 103	Responders: 35.4 [12.9], Nonresponders: 37.8 [10.3]	NR (Japan)	YES	IFX	No difference between genotypes
rs3819025	Urabe S. et al. (2015) [75]	CD: 103	Responders: 35.4 [12.9], Nonresponders: 37.8 [10.3]	NR (Japan)	YES	IFX	No difference between genotypes
**IL17F**							
rs763780	Urabe S. et al. (2015) [75]	CD: 103	Responders: 35.4 [12.9], Nonresponders: 37.8 [10.3]	NR (Japan)	YES	IFX	No difference between genotypes
rs12201582	Urabe S. et al. (2015) [75]	CD: 103	Responders: 35.4 [12.9], Nonresponders: 37.8 [10.3]	NR (Japan)	YES	IFX	No difference between genotypes
rs9382084	Urabe S. et al. (2015) [75]	CD: 103	Responders: 35.4 [12.9], Nonresponders: 37.8 [10.3]	NR (Japan)	YES	IFX	No difference between genotypes
rs722323	Urabe S. et al. (2015) [75]	CD: 103	Responders: 35.4 [12.9], Nonresponders: 37.8 [10.3]	NR (Japan)	YES	IFX	No difference between genotypes

rs1266828	Urabe S. et al. (2015) [75]	CD: 103	Responders: 35.4 [12.9], Nonresponders: 37.8 [10.3]	NR (Japan)	YES	IFX	No difference between genotypes
rs2294834	Urabe S. et al. (2015) [75]	CD: 103	Responders: 35.4 [12.9], Nonresponders: 37.8 [10.3]	NR (Japan)	YES	IFX	No difference between genotypes
**IL17RA**							
rs2270241	Urabe S. et al. (2015) [75]	CD: 103	Responders: 35.4 [12.9], Nonresponders: 37.8 [10.3]	NR (Japan)	YES	IFX	No difference between genotypes
rs2241042	Urabe S. et al. (2015) [75]	CD: 103	Responders: 35.4 [12.9], Nonresponders: 37.8 [10.3]	NR (Japan)	YES	IFX	No difference between genotypes
rs5748863	Urabe S. et al. (2015) [75]	CD: 103	Responders: 35.4 [12.9], Nonresponders: 37.8 [10.3]	NR (Japan)	YES	IFX	No difference between genotypes
rs5748864	Urabe S. et al. (2015) [75]	CD: 103	Responders: 35.4 [12.9], Nonresponders: 37.8 [10.3]	NR (Japan)	YES	IFX	No difference between genotypes
rs2241044	Urabe S. et al. (2015) [75]	CD: 103	Responders: 35.4 [12.9], Nonresponders: 37.8 [10.3]	NR (Japan)	YES	IFX	No difference between genotypes
rs2241049	Urabe S. et al. (2015) [75]	CD: 103	Responders: 35.4 [12.9], Nonresponders: 37.8 [10.3]	NR (Japan)	YES	IFX	No difference between genotypes
rs2229151	Urabe S. et al. (2015) [75]	CD: 103	Responders: 35.4 [12.9], Nonresponders: 37.8 [10.3]	NR (Japan)	YES	IFX	No difference between genotypes
rs2895332	Urabe S. et al. (2015) [75]	CD: 103	Responders: 35.4 [12.9], Nonresponders: 37.8 [10.3]	NR (Japan)	YES	IFX	No difference between genotypes
**IL17RC**							
rs7627880	Urabe S. et al. (2015) [75]	CD: 103	Responders: 35.4 [12.9], Nonresponders: 37.8 [10.3]	NR (Japan)	YES	IFX	No difference between genotypes
rs279545	Urabe S. et al. (2015) [75]	CD: 103	Responders: 35.4 [12.9], Nonresponders: 37.8 [10.3]	NR (Japan)	YES	IFX	No difference between genotypes
**IL1RN**							
rs315926	Tang et al. (2020) [36]	CD: 189	23 [18–28]	NR (China)	Yes	IFX	No difference between genotypes
rs315949	Tang et al. (2020) [36]	CD: 189	23 [18–28]	NR (China)	No	IFX	No difference between genotypes
rs396201	Tang et al. (2020) [36]	CD: 189	23 [18–28]	NR (China)	Yes	IFX	No difference between genotypes
rs77853995	Tang et al. (2020) [36]	CD: 189	23 [18–28]	NR (China)	Yes	IFX	No difference between genotypes
rs1688072	Tang et al. (2020) [36]	CD: 189	23 [18–28]	NR (China)	Yes	IFX	No difference between genotypes
rs117929702	Tang et al. (2020) [36]	CD: 189	23 [18–28]	NR (China)	Yes	IFX	No difference between genotypes
rs1665190	Tang et al. (2020) [36]	CD: 189	23 [18–28]	NR (China)	Yes	IFX	No difference between genotypes
rs55709272	Tang et al. (2020) [36]	CD: 189	23 [18–28]	NR (China)	Yes	IFX	No difference between genotypes
**IL1B**							
rs4848306	Salvador-Martín et al. (2019) A [32]	IBD:210, CD:147,	NR (<18 years)	NR (Spain)	NR	IFX, ADL	No difference between genotypes

		UC:63					
	Salvador-Martín et al. (2019) B [34]	CD: 132	27.4 [10.8-76.7]	NR (Spain)	Yes	IFX	No difference between genotypes
	Salvador-Martín et al. (2020) [19]	IBD:209, CD: 147, UC:62	10.6 [0.7–17.3]	NR (Spain)	Yes	IFX, ADL	No difference between genotypes
	Salvador-Martín et al. (2021) B [82]	IBD:154, CD:106, UC: 48	10.85 [1.5–17.3]	NR (Spain)	yes	IFX, ADL	No difference between genotypes
rs1143634	Lykowska-Szuber et al. (2023) [26]	CD: 196	30.65 [10.58]	NR (Poland)	Yes	IFX, ADL	No difference between genotypes
**IL23R**							
rs11465804	Nuij et al. (2017) [49]	CD: 411, UC:148, IC: 11	27 [5–79]	NR (Netherlands)	NR	IFX, ADL	No difference between genotypes
**LRRK2**							
rs11175593	Nuij et al. (2017) [49]	CD: 411, UC:148, IC: 11	27 [5–79]	NR (Netherlands)	NR	IFX, ADL	No difference between genotypes
**LTA**							
rs909253	Dideberg et al. (2006)B [108]	CD:214	38 [21-57]	Caucasian (NR)	NR	IFX	No difference between genotypes
	Stavrou et al. (2022) [87]	CD: 109	45 [11.27]	NR (Greece)	Yes	IFX, ADL	No difference between genotypes
rs2857713	Dideberg et al. (2006)B [108]	CD:214	38 [21-57]	Caucasian (NR)	NR	IFX	No difference between genotypes
rs5875327	Dideberg et al. (2006)B [108]	CD:214	38 [21-57]	Caucasian (NR)	NR	IFX	No difference between genotypes
rs1041981	Dideberg et al. (2006)B [108]	CD:214	38 [21-57]	Caucasian (NR)	NR	IFX	No difference between genotypes
rs746868	Dideberg et al. (2006)B [108]	CD:214	38 [21-57]	Caucasian (NR)	NR	IFX	No difference between genotypes
rs3093543	Dideberg et al. (2006)B [108]	CD:214	38 [21-57]	Caucasian (NR)	NR	IFX	No difference between genotypes
rs56285847	Perera et al. (2010) [81]	IBD: 105	NR	Caucasian (NR)	NR	Anti-TNF	No difference between genotypes
**LY96**							
rs11465996	Salvador-Martín et al. (2019) B [34]	CD: 132	27.4 [10.8-76.7]	NR (Spain)	Yes	IFX	No difference between genotypes
**MAP3K14**							
rs7222094	Salvador-Martín et al. (2019) A [32]	IBD:210, CD:147, UC:63	NR (<18 years)	NR (Spain)	NR	IFX, ADL	No difference between genotypes
	Salvador-Martín et al. (2019) B [34]	CD: 132	27.4 [10.8-76.7]	NR (Spain)	Yes	IFX	No difference between genotypes
	Salvador-Martín et al. (2020) [19]	IBD:209, CD: 147, UC:62	10.6 [0.7–17.3]	NR (Spain)	Yes	IFX, ADL	No difference between genotypes
	Salvador-Martín et al. (2021) B [82]	IBD:154, CD:106,	10.85 [1.5–17.3]	NR (Spain)	yes	IFX, ADL	No difference between genotypes

		UC: 48					
	Stavrou et al. (2022) [87]	CD: 109	45 [11.27]	NR (Greece)	Yes	IFX, ADL	No difference between genotypes
**MDR1**							
C3435T	Palmieri et al. (2005) [109]	CD: 478, UC: 468, control:450	CD: 43 [14], UC: 48 [15]	NR (Italy)	yes	IFX	No difference between genotypes
G2677T/A	Palmieri et al. (2005) [109]	CD: 478, UC: 468, control:450	CD: 43 [14], UC: 48 [15]	NR (Italy)	yes	IFX	No difference between genotypes
**MIR146A**							
rs2910164	Nani et al. (2023)[110]	CD: 103	43 [15.33]	NR (Greece)	yes	Anti-TNF	No difference between genotypes
	Papaconstantinou et al. (2017) [111]	CD: 107	Responders: 34.10[11.63], Partial responders: 32.23 [13.31], Primary nonresponders: 39.09 [15.60]	NR (Greece)	NR	IFX, ADL	No difference between genotypes
**MIR196A**							
rs11614913	Papaconstantinou et al. (2017) [111]	CD: 107	Responders: 34.10[11.63], Partial responders: 32.23 [13.31], Primary nonresponders: 39.09 [15.60]	NR (Greece)	NR	IFX, ADL	No difference between genotypes
**MIR221**							
rs113054794	Papaconstantinou et al. (2017) [111]	CD: 107	Responders: 34.10[11.63], Partial responders: 32.23 [13.31], Primary nonresponders: 39.09 [15.60]	NR (Greece)	NR	IFX, ADL	No difference between genotypes
**MIR224**							
rs188519172	Papaconstantinou et al. (2017) [111]	CD: 107	Responders: 34.10[11.63], Partial responders: 32.23 [13.31], Primary nonresponders: 39.09 [15.60]	NR (Greece)	NR	IFX, ADL	No difference between genotypes
**MIR155**							
rs767649	Nani et al. (2023)[110]	CD: 103	43 [15.33]	NR (Greece)	yes	Anti-TNF	No difference between genotypes
**NCF4**							
rs4821544	Nuij et al. (2017) [49]	CD: 411, UC:148, IC: 11	27 [5–79]	NR (Netherlands)	NR	IFX, ADL	No difference between genotypes
**NF-kB1**							
rs3774934	Dezelak et al. (2016) [50]	CD:79	26.6 [12.2]	NR (Slovenia)	NR	ADL	No difference between genotypes
rs4648011	Dezelak et al. (2016) [50]	CD:79	26.6 [12.2]	NR (Slovenia)	NR	ADL	No difference between genotypes
rs13117745	Dezelak et al. (2016) [50]	CD:79	26.6 [12.2]	NR (Slovenia)	NR	ADL	No difference between genotypes
rs7674004	Tang et al. (2020) [36]	CD: 189	23 [18–28]	NR (China)	Yes	IFX	No difference between genotypes
Haplotypes	Dezelak et al. (2016) [50]	CD:79	26.6 [12.2]	NR (Slovenia)	NR	ADL	No difference between genotypes

rs3774934<br>rs4648011<br>rs13117745							
rs4648141	Tang et al. (2020) [36]	CD: 189	23 [18–28]	NR (China)	Yes	IFX	No difference between genotypes
**NFKBIA**							
rs696	Dezelak et al. (2016) [50]	CD:79	26.6 [12.2]	NR (Slovenia)	NR	ADL	No difference between genotypes
**NOD2**							
rs2066844	Barreiro-de Acosta et al. (2010) [103]	CD: 24	40.5 [9.58]	NR (Spain)	NR	ADL	No difference between genotypes
	Nuij et al. (2017) [49]	CD: 411, UC:148, IC: 11	27 [5–79]	NR (Netherlands)	NR	IFX, ADL	No difference between genotypes
rs2066845	Barreiro-de Acosta et al. (2010) [103]	CD: 24	40.5 [9.58]	NR (Spain)	NR	ADL	No difference between genotypes
	Nuij et al. (2017) [49]	CD: 411, UC:148, IC: 11	27 [5–79]	NR (Netherlands)	NR	IFX, ADL	No difference between genotypes
rs2066847	Barreiro-de Acosta et al. (2010) [103]	CD: 24	40.5 [9.58]	NR (Spain)	NR	ADL	No difference between genotypes
	Nuij et al. (2017) [49]	CD: 411, UC:148, IC: 11	27 [5–79]	NR (Netherlands)	NR	IFX, ADL	No difference between genotypes
R702W, G908R and L1007fs	Lu et al. (2012) [106]	CD: 14	Remaining in remission: 30 [14-47], Lost remission: 26 [15-38]	Caucasian (Canada)	NR	IFX	No difference between genotypes
CARD15 (R702W/SNP8, G908R/SNP12, 3020insC/SNP13)	Mascheretti et al. (2002) A[112]	CD: 534	NR [18–65]	NR (Germany)	NR	IFX	No difference between genotypes
R702W	Vermeire et al. (2002) [113]	CD: 245	35 [28-44]	Caucasian (Belgium)	NR	IFX	No difference between genotypes
G908R	Vermeire et al. (2002) [113]	CD: 245	35 [28-44]	Caucasian (Belgium)	NR	IFX	No difference between genotypes
1007fs	Vermeire et al. (2002) [113]	CD: 245	35 [28-44]	Caucasian (Belgium)	NR	IFX	No difference between genotypes
**OSM**							
rs75951407	Tang et al. (2020) [36]	CD: 189	23 [18–28]	NR (China)	Yes	IFX	No difference between genotypes
**OSMR**							
rs357287	Tang et al. (2020) [36]	CD: 189	23 [18–28]	NR (China)	Yes	IFX	No difference between genotypes

rs595740	Tang et al. (2020) [36]	CD: 189	23 [18–28]	NR (China)	Yes	IFX	No difference between genotypes
rs13357358	Tang et al. (2020) [36]	CD: 189	23 [18–28]	NR (China)	Yes	IFX	No difference between genotypes
rs834006	Tang et al. (2020) [36]	CD: 189	23 [18–28]	NR (China)	No	IFX	No difference between genotypes
rs78776521	Tang et al. (2020) [36]	CD: 189	23 [18–28]	NR (China)	Yes	IFX	No difference between genotypes
rs115277096	Tang et al. (2020) [36]	CD: 189	23 [18–28]	NR (China)	Yes	IFX	No difference between genotypes
**RPL4**							
RPL4	Salvador-Martín et al. (2021) A [61]	IBD:38, CD:30, UC:8	10.5 [0.7–17]	NR (Spain)	NR	IFX, ADL	No difference between genotypes
**SLCO1B1**							
SLCO1B1	Wang et al. (2021) [54]	IBD: 51	39 [17.5]	NR (European countries)	Yes	IFX, ADL	No difference between genotypes
**TF**							
rs1799852	Repnik et al. (2016) [66]	CD: 68	NR	NR (Slovenia)	NR	ADL	No difference between genotypes
**TLR2**							
rs1816702	Salvador-Martín et al. (2020) [19]	IBD:209, CD: 147, UC:62	10.6 [0.7–17.3]	NR (Spain)	Yes	IFX, ADL	No difference between genotypes
rs3804099	Salvador-Martín et al. (2020) [19]	IBD:209, CD: 147, UC:62	10.6 [0.7–17.3]	NR (Spain)	Yes	IFX, ADL	No difference between genotypes
rs3804099	Salvador-Martín et al. (2021) B [82]	IBD:154, CD:106, UC: 48	10.85 [1.5–17.3]	NR (Spain)	yes	IFX, ADL	No difference between genotypes
**TLR4**							
rs5030728	Hu et al. (2021) [41]	CD: 62	11.00 [8.00-12.41]	NR (china)	NR	IFX	No difference between genotypes
rs5030728	Salvador-Martín et al. (2019) B [34]	CD: 132	27.4 [10.8-76.7]	NR (Spain)	Yes	IFX	No difference between genotypes
rs5030728	Salvador-Martín et al. (2020) [19]	IBD:209, CD: 147, UC:62	10.6 [0.7–17.3]	NR (Spain)	Yes	IFX, ADL	No difference between genotypes
rs5030728	Stavrou et al. (2022) [87]	CD: 109	45 [11.27]	NR (Greece)	Yes	IFX, ADL	No difference between genotypes
rs4986790	Barreiro-de Acosta et al. (2010) [103]	CD: 24	40.5 [9.58]	NR (Spain)	NR	ADL	No difference between genotypes
**TLR9**							
rs352139	Salvador-Martín et al. (2019) A[32]	IBD:210, CD:147, UC:63	NR (<18 years)	NR (Spain)	NR	IFX, ADL	No difference between genotypes
rs352139	Salvador-Martín et al. (2019) B [34]	CD: 132	27.4 [10.8-76.7]	NR (Spain)	Yes	IFX	No difference between genotypes

	Salvador-Martín et al. (2020) [19]	IBD:209, CD: 147, UC:62	10.6 [0.7–17.3]	NR (Spain)	Yes	IFX, ADL	No difference between genotypes
	Salvador-Martín et al. (2021) B [82]	IBD:154, CD:106, UC: 48	10.85 [1.5–17.3]	NR (Spain)	yes	IFX, ADL	No difference between genotypes
**TNF**							
rs1799964	Dideberg et al. (2006) A[101]	CD: 222	21 [11-31]	Caucasian (NR)	Yes	IFX	No difference between genotypes
rs1800630	Dideberg et al. (2006) A[101]	CD: 222	21 [11-31]	Caucasian (NR)	Yes	IFX	No difference between genotypes
rs4248158	Dideberg et al. (2006) A[101]	CD: 222	21 [11-31]	Caucasian (NR)	Yes	IFX	No difference between genotypes
rs4248160	Dideberg et al. (2006)A [101]	CD: 222	21 [11-31]	Caucasian (NR)	Yes	IFX	No difference between genotypes
rs4248161	Dideberg et al. (2006) A [101]	CD: 222	21 [11-31]	Caucasian (NR)	Yes	IFX	No difference between genotypes
rs1800750	Dideberg et al. (2006) A [101]	CD: 222	21 [11-31]	Caucasian (NR)	Yes	IFX	No difference between genotypes
rs3093661	Dideberg et al. (2006) A [101]	CD: 222	21 [11-31]	Caucasian (NR)	Yes	IFX	No difference between genotypes
rs4645839	Dideberg et al. (2006) A [101]	CD: 222	21 [11-31]	Caucasian (NR)	Yes	IFX	No difference between genotypes
rs1800610	Dideberg et al. (2006) A[101]	CD: 222	21 [11-31]	Caucasian (NR)	Yes	IFX	No difference between genotypes
rs4645841	Dideberg et al. (2006) A [101]	CD: 222	21 [11-31]	Caucasian (NR)	Yes	IFX	No difference between genotypes
rs1799769	Dideberg et al. (2006) A[101]	CD: 222	21 [11-31]	Caucasian (NR)	Yes	IFX	No difference between genotypes
rs4645842	Dideberg et al. (2006) A [101]	CD: 222	21 [11-31]	Caucasian (NR)	Yes	IFX	No difference between genotypes
rs3093662	Dideberg et al. (2006) A [101]	CD: 222	21 [11-31]	Caucasian (NR)	Yes	IFX	No difference between genotypes
rs3093664	Dideberg et al. (2006) A[101]	CD: 222	21 [11-31]	Caucasian (NR)	Yes	IFX	No difference between genotypes
rs3093665	Dideberg et al. (2006) A[101]	CD: 222	21 [11-31]	Caucasian (NR)	Yes	IFX	No difference between genotypes
-308 G>A	Louis et al. (2002)[114]	CD: 214	Luminal disease: 35.9 [11.6], Fistulizing disease: 37.8 [10.9]	Caucasian (Belgium)	NR	IFX	No difference between genotypes
	Papamichael et al. (2011)[97]	CD: 106	Complete responders: 32 [14.78], Partial responders: 27.33 [13.98], Primary nonresponders: 27.67 [16.58]	NR (Greece)	NR	IFX	No difference between genotypes
	Perera et al. (2010) [81]	IBD: 105	NR	Caucasian (NR)	NR	Anti-TNF	No difference between genotypes
	Tomita et al. (2010)[60]	CD: 41	30 [18-54]	NR (Japan)	NR	IFX	No difference between genotypes
-857 C/T	Papamichael et al. (2011) [97]	CD: 106	Complete responders: 32 [14.78], Partial responders: 27.33 [13.98], Primary nonresponders: 27.67 [16.58]	NR (Greece)	NR	IFX	No difference between genotypes
	Perera et al. (2010) [81]	IBD: 105	NR	Caucasian (NR)	NR	Anti-TNF	No difference between genotypes
rs1800750	Perera et al. (2010) [81]	IBD: 105	NR	Caucasian (NR)	NR	Anti-TNF	No difference between genotypes
rs1799724	Cheli et al. (2023)[46]	CD: 56, UC: 23	13 [2.5]	NR (Italy)	Yes	IFX, ADL	No difference between genotypes

	Dideberg et al. (2006) A[101]	CD: 222	21 [11-31]	Caucasian (NR)	Yes	IFX	No difference between genotypes
rs1800629	Curci et al. (2021)[38]	CD: 50, UC: 26	14.3 [12.3-16.3]	NR (Italy)	Yes	IFX	No difference between genotypes
	Dideberg et al. (2006) A[101]	CD: 222	21 [11-31]	Caucasian (NR)	Yes	IFX	No difference between genotypes
	Matsuoka et al. (2018) [88]	CD: 121	37.5 [9.5]	NR (Japan)	yes	IFX	No difference between genotypes
	Salvador-Martín et al. (2019) A [32]	IBD:210, CD:147, UC:63	NR (<18 years)	NR (Spain)	NR	IFX, ADL	No difference between genotypes
	Salvador-Martín et al. (2019) B [34]	CD: 132	27.4 [10.8-76.7]	NR (Spain)	Yes	IFX	No difference between genotypes
	Salvador-Martín et al. (2020) [19]	IBD:209, CD: 147, UC:62	10.6 [0.7–17.3]	NR (Spain)	Yes	IFX, ADL	No difference between genotypes
	Salvador-Martín et al. (2021) B [82]	IBD:154, CD:106, UC: 48	10.85 [1.5–17.3]	NR (Spain)	yes	IFX, ADL	No difference between genotypes
rs361525	Cheli et al. (2023) [46]	CD: 56, UC: 23	13 [2.5]	NR (Italy)	Yes	IFX, ADL	No difference between genotypes
	Dideberg et al. (2006) A[101]	CD: 222	21 [11-31]	Caucasian (NR)	Yes	IFX	No difference between genotypes
	Lopez-hernandez et al. (2013)[89]	CD:54, UC:28	Below 18 years: CD(7, 13%), UC(1, 4%); Between 18 - 40 years: CD (28, 52%), UC (13, 46%); Above 40 years: CD (19, 35%), UC: (14, 50%)	NR (Spain)	NR	IFX, ADL	No difference between genotypes
	Netz et al. (2017)[29]	CD:121	41.6 [95%CI: 39.2-44.0]	Caucasian 93% (USA)	yes	Anti-TNF	No difference between genotypes
	Papamichael et al. (2011) [97]	CD: 106	Complete responders: 32 [14.78], Partial responders: 27.33 [13.98], Primary nonresponders: 27.67 [16.58]	NR (Greece)	NR	IFX	No difference between genotypes
	Matsuoka et al. (2018) [88]	CD: 121	37.5 [9.5]	NR (Japan)	yes	IFX	No difference between genotypes
	Salvador-Martín et al. (2019) A [32]	IBD:210, CD:147, UC:63	NR (<18 years)	NR (Spain)	NR	IFX, ADL	No difference between genotypes
	Salvador-Martín et al. (2019) B [34]	CD: 132	27.4 [10.8-76.7]	NR (Spain)	Yes	IFX	No difference between genotypes
	Salvador-Martín et al. (2020) [19]	IBD:209, CD: 147, UC:62	10.6 [0.7–17.3]	NR (Spain)	Yes	IFX, ADL	No difference between genotypes
	Salvador-Martín et al. (2021) B [82]	IBD:154, CD:106,	10.85 [1.5–17.3]	NR (Spain)	yes	IFX, ADL	No difference between genotypes

		UC: 48					
**TNFAIP3**							
rs6927172	Salvador-Martín et al. (2019) B [34]	CD: 132	27.4 [10.8-76.7]	NR (Spain)	Yes	IFX	No difference between genotypes
	Salvador-Martín et al. (2020) [19]	IBD:209, CD: 147, UC:62	10.6 [0.7–17.3]	NR (Spain)	Yes	IFX, ADL	No difference between genotypes
	Salvador-Martín et al. (2021) B [82]	IBD:154, CD:106, UC: 48	10.85 [1.5–17.3]	NR (Spain)	yes	IFX, ADL	No difference between genotypes
**TNFRSF1A**							
rs4149570	Hu et al. (2021) [41]	CD: 62	11.00 [8.00-12,41]	NR (china)	NR	IFX	No difference between genotypes
	Matsukura et al. (2008) [90]	CD: 80	Responders: 29.9 [7.5], Nonresponders: 33.7 [9.7]	Asian (Japan)	yes	IFX	No difference between genotypes
	Salvador-Martín et al. (2019) A [32]	IBD:210, CD:147, UC:63	NR (<18 years)	NR (Spain)	NR	IFX, ADL	No difference between genotypes
	Salvador-Martín et al. (2019) B [34]	CD: 132	27.4 [10.8-76.7]	NR (Spain)	Yes	IFX	No difference between genotypes
	Salvador-Martín et al. (2020) [19]	IBD:209, CD: 147, UC:62	10.6 [0.7–17.3]	NR (Spain)	Yes	IFX, ADL	No difference between genotypes
	Salvador-Martín et al. (2021) B [82]	IBD:154, CD:106, UC: 48	10.85 [1.5–17.3]	NR (Spain)	yes	IFX, ADL	No difference between genotypes
	Steenholdt et al. (2012) [22]	CD:104	Primary nonresponse: 22 [19–42], Loss of response: 24 [20–38], Maintained remission: 25 [20–32]	Caucasian (NR)	Yes	IFX	No difference between genotypes
rs767455	Matsuoka et al. (2018) [88]	CD: 121	37.5 [9.5]	NR (Japan)	yes	IFX	No difference between genotypes
	Medrano et al. (2014) [21]	CD:297	Nonresponders:43.1 [1.6], Responders: 39.9 [0.8]	Caucasian (Spain)	Yes	IFX	No difference between genotypes
	Salvador-Martín et al. (2019) A [32]	IBD:210, CD:147, UC:63	NR (<18 years)	NR (Spain)	NR	IFX, ADL	No difference between genotypes
	Salvador-Martín et al. (2019) B [34]	CD: 132	27.4 [10.8-76.7]	NR (Spain)	Yes	IFX	No difference between genotypes
	Salvador-Martín et al. (2020) [19]	IBD:209, CD: 147, UC:62	10.6 [0.7–17.3]	NR (Spain)	Yes	IFX, ADL	No difference between genotypes
	Salvador-Martín et al. (2021) B [82]	IBD:154, CD:106,	10.85 [1.5–17.3]	NR (Spain)	yes	IFX, ADL	No difference between genotypes

		UC: 48					
rs4149584	Steenholdt et al. (2012) [22]	CD:104	Primary nonresponse: 22 [19–42], Loss of response: 24 [20–38], Maintained remission: 25 [20–32]	Caucasian (NR)	Yes	IFX	No difference between genotypes
rs4149569	Steenholdt et al. (2012) [22]	CD:104	Primary nonresponse: 22 [19–42], Loss of response: 24 [20–38], Maintained remission: 25 [20–32]	Caucasian (NR)	Yes	IFX	No difference between genotypes
**TNFRSF1B**							
rs3397	Salvador-Martín et al. (2019) A [32]	IBD:210, CD:147, UC:63	NR (<18 years)	NR (Spain)	NR	IFX, ADL	No difference between genotypes
	Salvador-Martín et al. (2019) B [34]	CD: 132	27.4 [10.8-76.7]	NR (Spain)	Yes	IFX	No difference between genotypes
	Salvador-Martín et al. (2020) [19]	IBD:209, CD: 147, UC:62	10.6 [0.7–17.3]	NR (Spain)	Yes	IFX, ADL	No difference between genotypes
rs5746053	Lykowska-Szuber et al. (2023) [26]	CD: 196	30.65 [10.58]	NR (Poland)	Yes	IFX, ADL	No difference between genotypes
rs5746054	Lykowska-Szuber et al. (2023) [26]	CD: 196	30.65 [10.58]	NR (Poland)	Yes	IFX, ADL	No difference between genotypes
rs1061622	Matsukura et al. (2008) [90]	CD: 80	Responders: 29.9 [7.5], Nonresponders: 33.7 [9.7]	Asian (Japan)	yes	IFX	No difference between genotypes
	Salvador-Martín et al. (2019) A [32]	IBD:210, CD:147, UC:63	NR (<18 years)	NR (Spain)	NR	IFX, ADL	No difference between genotypes
	Salvador-Martín et al. (2019) B [34]	CD: 132	27.4 [10.8-76.7]	NR (Spain)	Yes	IFX	No difference between genotypes
	Salvador-Martín et al. (2020) [19]	IBD:209, CD: 147, UC:62	10.6 [0.7–17.3]	NR (Spain)	Yes	IFX, ADL	No difference between genotypes
	Salvador-Martín et al. (2021) B [82]	IBD:154, CD:106, UC: 48	10.85 [1.5–17.3]	NR (Spain)	yes	IFX, ADL	No difference between genotypes
rs1061624	Hu et al. (2021) [41]	CD: 62	11.00 [8.00-12,41]	NR (china)	NR	IFX	No difference between genotypes
	Lykowska-Szuber et al. (2023) [26]	CD: 196	30.65 [10.58]	NR (Poland)	Yes	IFX, ADL	No difference between genotypes
	Salvador-Martín et al. (2019) A [32]	IBD:210, CD:147, UC:63	NR (<18 years)	NR (Spain)	NR	IFX, ADL	No difference between genotypes
	Salvador-Martín et al. (2020) [19]	IBD:209, CD: 147, UC:62	10.6 [0.7–17.3]	NR (Spain)	Yes	IFX, ADL	No difference between genotypes
	Salvador-Martín et al. (2021) B [82]	IBD:154,	10.85 [1.5–17.3]	NR (Spain)	yes	IFX, ADL	No difference between genotypes

		CD:106, UC: 48					
196/Arg	Mascheretti et al. (2004) B [115]	CD: 99, Control: 444	NR [18–65]	NR (Germany)	NR	IFX	No difference between genotypes
rs976881	Steenholdt et al. (2012) [22]	CD:104	Primary nonresponse: 22 [19–42], Loss of response: 24 [20–38], Maintained remission: 25 [20–32]	Caucasian (NR)	Yes	IFX	No difference between genotypes
rs5746026	Steenholdt et al. (2012) [22]	CD:104	Primary nonresponse: 22 [19–42], Loss of response: 24 [20–38], Maintained remission: 25 [20–32]	Caucasian (NR)	Yes	IFX	No difference between genotypes
**TNFR2**							
587G	Pierik et al. (2004) [91]	CD: 344, UC: 152	42.4 [18–76]	NR (Belgium)	yes	IFX	No difference between genotypes
rs976881	Matsuoka et al. (2018) [88]	CD: 121	37.5 [9.5]	NR (Japan)	yes	IFX	No difference between genotypes
rs1061622	Matsuoka et al. (2018) [88]	CD: 121	37.5 [9.5]	NR (Japan)	yes	IFX	No difference between genotypes
**TRAF3IP2**							
rs10872070	Urabe S. et al. (2015) [75]	CD: 103	Responders: 35.4 [12.9], Nonresponders: 37.8 [10.3]	NR (Japan)	YES	IFX	No difference between genotypes
rs6941014	Urabe S. et al. (2015) [75]	CD: 103	Responders: 35.4 [12.9], Nonresponders: 37.8 [10.3]	NR (Japan)	YES	IFX	No difference between genotypes
rs1040383	Urabe S. et al. (2015) [75]	CD: 103	Responders: 35.4 [12.9], Nonresponders: 37.8 [10.3]	NR (Japan)	YES	IFX	No difference between genotypes
rs9374263	Urabe S. et al. (2015) [75]	CD: 103	Responders: 35.4 [12.9], Nonresponders: 37.8 [10.3]	NR (Japan)	YES	IFX	No difference between genotypes
rs2075966	Urabe S. et al. (2015) [75]	CD: 103	Responders: 35.4 [12.9], Nonresponders: 37.8 [10.3]	NR (Japan)	YES	IFX	No difference between genotypes
**UGT1A1**							
UGT1A1	Wang et al. (2021) [54]	IBD: 51	39 [17.5]	NR (European countries)	Yes	IFX, ADL	No difference between genotypes
**VKORC1**							
VKORC1	Wang et al. (2021) [54]	IBD: 51	39 [17.5]	NR (European countries)	Yes	IFX, ADL	No difference between genotypes
**Unknown**							
rs4411591	Thomas et al. (2014) [95]	CD: 126	Complete responders: 28.42 [12.85] Partial responders: 26.65 [14.21] Primary non-responders:27.32 [13.88]	NR (Greece)	NR	IFX	No difference between genotypes

ADL: Adalimumab, anti.TNF: anti Tumor Necrosis Factor, CD: Crohn Disease, HWE: Hardy-Weinberg Equilibrium, IBD: Inflammatory Bowel Disease, IC: Indeterminate Colitis, IFX: Infliximab, NR: Not Reported, SD: Standard Deviation, SNP: Single Nucleotide Polymorphisms, USA: United States of America, UC: Ulcerative Colitis.
